# Supplementary material for: Metacognition biases information seeking in assessing ambiguous news
Source: Commun Psychol. 2024 Dec 19;2:122. doi: 10.1038/s44271-024-00170-w (PMC11659316; doi:10.1038/s44271-024-00170-w)
Supplement: Supplementary file 2 — Supplementary Information [file 44271_2024_170_MOESM2_ESM.pdf]

Supplementary Information

Index

Index 1

I. Supplementary Methods I: Stimuli selection........ 2

1. Stimuli selection procedure 2
2. Stimuli selection instructions (translated from French) 3

II. Supplementary Methods II: Main experiment task instructions (translated from French)... 4

1. Part 1 4
2. Part 2 5
3. Comprehension questionnaire........10

III. Supplementary Methods III: Presentation of the organizations (translated from French)..18

1. Democracy-related organizations 18
2. Ecology-related organizations 19
3. Social justice – related organizations 20

IV. Supplementary Methods IV: Post-task questionnaires (translated from French) 21

1. Epistemic Curiosity questionnaire........21
2. Exposition to information........21
3. Perceived percentage of fake news 22
4. Manipulation check 22

V. Supplementary Methods V: Sample size and data quality assessment 23

1. Sentiment analysis 23
2. Power simulation........23
3. Distributions of success in estimating veracity 24
4. Distributions of demand for further information and related WTP 24

VI. Supplementary Methods VI: Behavioral analyses 25

1. Chance-level veracity estimation 25
2. Mixed Linear Models of veracity estimation 26
3. Comparison of Bayesian Mixed Linear Models of veracity estimation 35
4. Mixed Linear Models of metacognitive abilities 37
5. Mixed Linear Models of demand for further information 42
6. Moderated Mediation Model 45

Supplementary References......46

I. Supplementary Methods I: Stimuli selection

1. Stimuli selection procedure

To select the stimuli we followed the practical guide of Pennycook and colleagues for behavioral research on fake news and misinformation [1]. We first designed a set of 210 true and false brief news (114 false news, 96 true news). The maximal length of each news was 140 characters, spaces included. We restricted the nature of these news to information with a cognitive utility, that is, factual information which content refers to concepts that individuals often think of with a capacity to alter their understanding of the state of the world. We avoided ego-relevant stimuli and stimuli that could elicit affects or have short term consequences on participants' daily decision-making. The selected brief news described events or statements about ecology, social justice and democracy – three keys themes that gained momentum at time of the experiment as hot topics but did not directly concerned participants' health (as would news related to the COVID-19 pandemic). Some of the brief news were directly taken from the French fake news debunk platforms *Les Décodeurs du Monde*, *AFP Factcheck* and *Libération Checknews* from the period 2017-2020. Others have been fabricated from content on these platforms.

We then completed a pretest to ensure that our stimuli varied in content imprecision and met agreement regarding the themes of the news. We planned to keep 96 counterbalanced news after the pretest, with no statistical difference in content imprecision and capacity to make consensus on the related theme between the set of true news and the set of false news. The pretest ran on Testable.org and was rewarded $7. A total of fifty-five independent raters (F=33, M=22; mean age=26.2, SD age=4.78) evaluated the news. Five groups of 11 French-speaking raters evaluated each a subset of 42 true and false news out of the 210. They evaluated each the stimuli content imprecision (M=5.28, SD=1.32), desirability (M=6.28, SD=1.27), propensity to polarize (M=6.41, SD=1.62) and themes.

Raters had to answer the five following questions: 'On a scale of 0 to 10, to what extent would you say that the content of this information lacks precision? (0 = not at all imprecise - 10 = very imprecise)'; 'On a scale of 0 to 10, to what extent would you like to know more about the content of this information? (0 = I would not at all want to know more at all - 10 = I would very much want to know more); 'On a scale of 0 to 10, how divisive and how likely to divide opinion do you think the content of this information is? (0 = not at all likely to divide opinion - 10 = very likely to divide opinion); 'Which theme do you think best fits the content of this information: Ecology, Democracy, Social justice, Health, Economy, Education, Identity, Security, Travel, Freedom, None of the above'; 'What other theme do you think would best fit the content of this information?'. Themes other than Ecology, Democracy or Social Justice were distractors in the forced-choice question. Instructions were presented as follows.

For each theme and each veracity level, we kept the 16 news that reached the highest agreement on theme, determined by the frequency of each theme. As a result, the final set of stimuli included 96 counterbalanced true and false news that were categorized as either democracy-related, ecology-related or social justice-related. ICC (ICC2k) estimates and their 95% confident intervals for the

measures of content imprecision, propensity to polarize and desirability were calculated using the package *psych* for R based on a mean-rating (k = 3), absolute-agreement, 2-way mixed-effects model. The average random raters' correlation coefficient was equal to 0.54 for content imprecision (95% CI [0.4, 0.66]), 0.81 for propensity to polarize (95% CI [0.75, 0.87], *p*<.001) and 0.59 for desirability (95% CI [0.46,0 .7], *p*<.001). ICC values below 0.5 usually indicate poor reliability, whereas values between 0.5 and 0.75 indicate moderate reliability, values from 0.75 to 0.9 indicate good reliability, and values above 0.90 indicate excellent reliability.[2]

No difference was found between true (M=5.53, SD=1.24) and false (M=5.17, SD=1.25) news content imprecision distributions ($ranksum$, p=0.09), nor between true (M=6.24, SD=1.57) and false (M=6.55, SD=1.55) news propensity to polarize distributions ($ranksum$, p=0.16). Overall, content imprecision was balanced between false and true news, and both were equally divisive among raters. We also found a (medium) correlation between the stimuli desirability, as evaluated by the raters, and the choices of the participants in the experiment to receive more information about the brief news (Rhô=0.349; *p*<0.001).

Moreover, we performed a sentiment analysis on the content of each stimulus. Sentiment analysis is a natural language processing technique used to determine whether the emotional valence of data is negative, neutral or positive. We used a multilingual XLM-roBERTa-base model sequence classifier based on Cardiff NLP Group sentiment classification model[3]. The model was trained on ~198M tweets and fine-tuned for sentiment analysis on eight languages, including French. The analysis was performed with the Python package *transformers* [4]. Out of the 96 stimuli, 90 had a probability score higher than 0.5 for the neutral dimension. In other words, 93.75% of the news were predominantly categorized as emotionally neutral.

2. Stimuli selection instructions (translated from French)

Welcome to this session.

Please, turn off your phone.

You will be participating in a session consisting of 42 periods.

This session will last approximately 40 minutes.

In each period, you will see a short piece of information. For each brief news, you will be asked to answer five questions. The first four questions will be, for each brief, mandatory.

The brief news will be different in each period.

Some of the brief news are from the French written press from the years 2017-2020. Others have been fabricated.

Unless specified otherwise, their content is recent, topical and concerns the French territory.

Please read each brief and each question carefully, then take the time you need to answer.

II. Supplementary Methods II: Main experiment task instructions (translated from French)

Welcome to this session.

Please, turn off your phone.

Then, please read the following instructions carefully. They will give you all the information you need to participate in this session.

Please note: The Testable.org platform will expire suspicious activity (window change, inactivity, excessively long duration). Once an entry has expired, it is no longer possible to re-enter.

Please click OK to continue.

You will receive **$9** for participating in the session.

Depending on your decisions, you may earn Experimental Currency Units (ECU). These ECU will be converted into US Dollars at a ratio of 100 ECU = $2.

At the end of the session, you will receive the monetary amount equivalent to the conversion of the ECU you earned plus $9 for your participation.

This session is composed of **two parts. Your answers during these two parts will be anonymous.**

They may be used in a future experiment. Nothing will identify you.

The instructions you will receive now are those for the first part.

You will receive the instructions for the second part at the end of the first part.

All the instructions will be displayed on your screen.

1. Part 1

The first part of this experiment involves evaluating 12 different organizations using six criteria. You will evaluate each criterion using the information you are given. You can also use your personal knowledge.

You will first learn about the organization to be evaluated and a summary of their purpose from their website, using the original wording, as in the example below:

La Pétanque Carryenne

La Pétanque Carryenne has existed in the commune of Carry-le-Rouet for over sixty years. In the past, competitions were held on the market square and at the *Family* (no longer in existence). No fewer than 10 presidents have passed on the torch to ensure the continued practice of the *jeu de boules*, both *pétanque* and *jeu provençal*.

The current *La Pétanque Carryenne* ground was built in the 1970s, and the number of members has grown steadily. The club has had the honor of hosting three French veteran *pétanque* champions and winners of the prestigious *La Marseillaise à Pétanque* world competition.

Today, the club has almost three hundred members and, for the past three years, a *pétanque* school to pass on the passion to new generations, and as the club's motto says: "Here, *pétanque* is more than a game, it's a religion! It's in an exceptional setting, in the heart of the town and just a stone's throw from the Big Blue that the *Carryens* will welcome you every day for exciting, friendly games.

Organizations rating

Then, you will evaluate this organization by answering the following 6 questions using a 7-point response scale:

- How familiar is this organization to you?
- How close do you feel the values of this organization are to your own?
- How much do you like this organization?
- How familiar do you think this organization is to those closest to you?
- How close do you think your family and friends feel that the values of this organization are to their own?
- How much do you think your loved ones would appreciate this organization?

For example, below, 1 means that the organization is 'Not familiar at all' and 7 means that the organization is 'Very familiar':

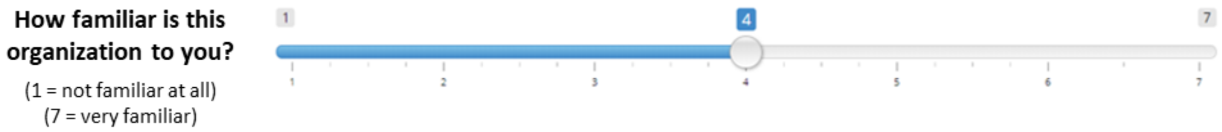

These evaluations are done privately.

Some of them may be shared with other participants in a future experiment, but they will not be associated with any element that could identify you.

The first part will now start

Throughout the session, use your computer mouse to respond.

When you are ready, please click OK.

First part completed

The first part is now complete. The instructions for the second part will begin.

Please read them carefully.

They will outline the rest of the session and explain how you will be compensated based on your performance.

To view them, please press "OK".

2. Part 2

At the beginning of each period, you will see a brief news. These briefs will each be different and come from different media. For each brief, there is a supplementary information.

The supplementary information consists of an investigative file. Their content is related to the content of the brief and can be related to other information around the content of the brief.

Please note: briefs can be true or false. Some of the stories you will be exposed to and play with will be false information.

False information has been fabricated. The non-fabricated news briefs are from the French print media over the period 2017 - 1st quarter 2020. Unless specified otherwise, their content is recent, topical and concerns the French territory.

Each period consists of two steps: the evaluation of the veracity of the news item and the decision to receive or not more information.

First step: news evaluation

After the brief information is displayed, you will be asked to rate whether its content is true or false as follows:

"In your opinion, what is the number of chances out of 100 that this brief is true or false?".

To answer, you will drag the slider below the question, as in the example below:

- To answer that the content of the brief is false, you will drag the slider between the 0 and -100 bounds.

- To answer that the content of the brief is true, you will drag the cursor between the 0 and +100 bounds.

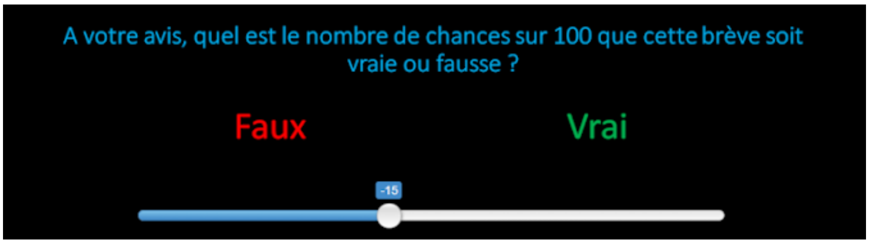

First step: robots to help you answer

To help you answer this question, there will be "bots" available during this step.

There are 100 different robots. Each robot has a certain accuracy level.

This accuracy level is the number of chances out of 100 that the robot correctly evaluates the brief news.

This number is an integer between 1 and 100. Each robot has a different accuracy level than the other robots.

That is, there is one robot that has a 1 in 100 chance of answering correctly, there is one robot that has a 2 in 100 chance of answering correctly, and so on until the 100th robot that has a 100 in 100 chance of answering correctly.

A robot that has 75 chances out of 100 to answer correctly will give a correct answer 75% of the time and will give a wrong answer 25% of the time.

At each period, the computer will draw one robot from the 100 robots. All robots have the same chance of being drawn. You will not know which one has been drawn and the robot will change randomly each period.

When providing your answer about the veracity of the brief, you will have to specify which bots you would let answer for you.

To do this, you will first move the cursor between the bounds corresponding to your assessment of veracity ('False'/'True'). Then, you decide how confident you are in your answer by choosing an accuracy threshold with the slider, as in the following example:

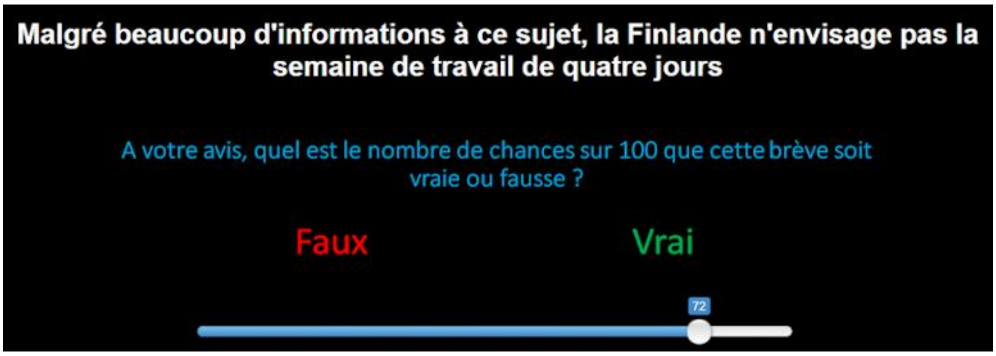

The accuracy threshold determines the threshold at which you would prefer the program to consider a robot's response rather than yours. The program will take the robot's answer into **account if and only if** its accuracy level is higher than the threshold you have chosen.

Thus, you will choose your accuracy threshold so that, for any robot with an **accuracy level less than or equal to your threshold**, you would prefer it to be **your answer** that the program takes into account.

For example:

- If you choose 75 as your threshold and the randomly selected robot has an accuracy level of 90, the program will consider that robot's answer. The robot will have a 90 out of 100 chance of giving the right answer.

- If you choose 75 as the accuracy threshold and the randomly selected robot has an accuracy level of 20, the program will consider your answer.

Thus, it is in your best interest to truly state how correct you think your answer is.

The less certain you are of your answer, the better it is to choose a low threshold.

That is, if you think there is a 25 in 100 chance that your answer is correct, it is better to give a threshold of 25.

In the following example, there are two decisions:

1) The participant answers that the content of the brief is true. He/she does this by positioning the cursor between the bounds 0 and 100. These bounds correspond to the answer 'True' to the question about veracity.

2) He/she would prefer that the program considers the robot's answer if and only if the robot drawn has more than 42 chances out of 100 to answer correctly. Therefore, he/she thinks that there is a 42 out of 100 chance that the brief is true.

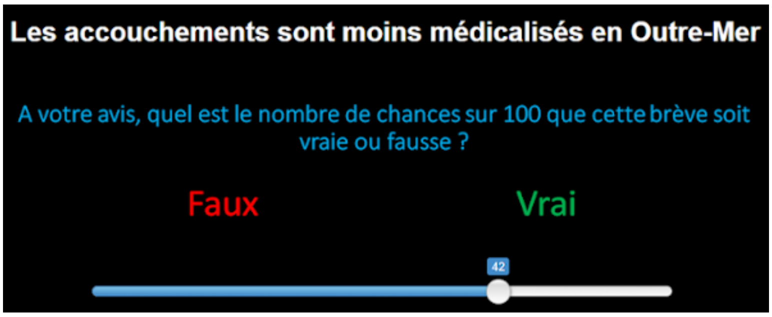

Step 1: Compensation based on your performance.

Eight of your assessments will be **randomly** selected at the end of the session.

Your compensation will be determined based only on the randomly selected assessments.

Each correct randomly selected assessment will earn you 50 ECU.

Please note: 0 is not a valid answer. Any time you answer with 0 will automatically be considered a failure.

Step 2: Receiving more information

In each period, once you have evaluated the brief, you will be asked **whether you would like to receive or not more information** about the content of the brief. You will do this by positioning the cursor on the desired answer, as in the example below.

Eight periods will be drawn at the end of the session, each with the same number of chances. Your choices in these eight periods will help determine your earnings.

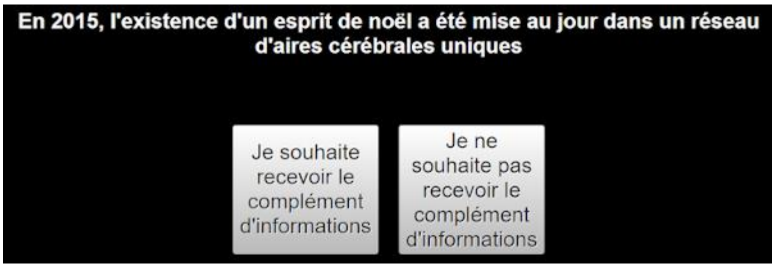

Step 2: Willingness to receive or avoid more information

Once you have chosen, you will be asked how many ECU you would be willing to deduct from your initial endowment to have your decision implemented.

You start the session with 200 ECU.

If you have indicated that you would like to receive more information, you will be asked how many ECU you would be willing to deduct from your initial endowment to **receive** the additional information.

You will be asked to choose how many ECU, between 0 and 25 ECU, you would be willing to deduct from your initial endowment.

To indicate the desired value, you will move the cursor between the values 0 and 25, as in the example below.

Attention: the position of the buttons "I wish to receive the additional information" and "I do not wish to receive the additional information" will be modified during the second half of the session.

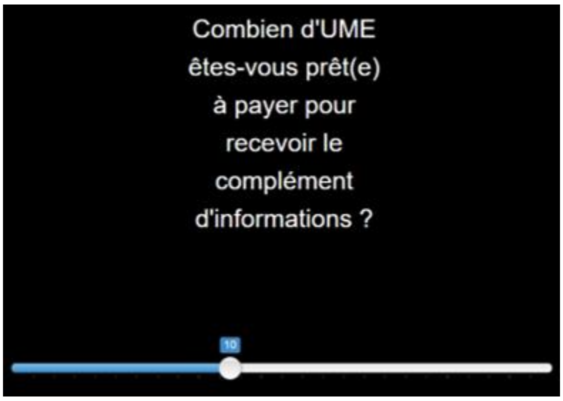

If you have indicated that you would not like to receive more information, you will be asked how many ECU you would be willing to deduct from your initial endowment to not receive the top-up.

You will be asked to choose how many ECU between 0 and 25 ECU you would be willing to deduct from your initial endowment.

To indicate the desired value, you will move the cursor between the values 0 and 25, as in the example below:

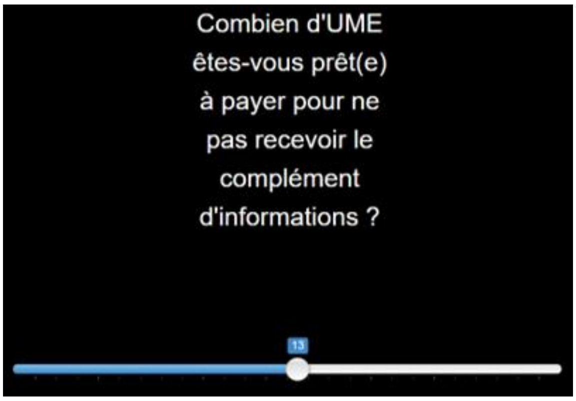

Step 2: Receiving more information and payment based on your decisions

Eight of your decisions, separate from the eight briefs evaluation periods, will be **randomly** selected at the end of the session.

They will determine whether or not you receive more information at the end of the session.

For each decision period selected at random, we will draw a number Y between 0 and 25. Your choice will be implemented if the drawn number of ECU Y is **less than or equal** to the number X of ECU you are willing to pay. **Your initial allocation will then decrease by Y ECU, the number drawn at random.**

Choosing 15 ECU means that you are willing to pay up to 15 ECU for your decision to be implemented.

Step 2: Implementing your decisions

Depending on your choices during the randomly selected periods at the end of the session, you will actually receive additional information in the days following your participation.

That is to say, there is an information supplement for each brief. The supplementary information consists of an investigation file. Their content is related to the content of the news item and can also be related to other information around the content of the news item.

For each randomly selected period, your choice of reception will be retained and implemented. The implementation consists in deducting from your initial endowment the amount of ECU and in sending you by email the files associated with the selected information.

The implementation of your choice will depend on your choice (to receive or not to receive) and the amount of ECU you are willing to pay.

Your decisions about whether or not to receive more information will therefore impact your reward at the end of the task in terms of information and ECUs received.

Calculating your earnings

A. Information evaluation:

At the end of the experiment, eight periods will be drawn from the 48 periods in Part 2. Each period will have an equal chance of being drawn.

A correct answer, either yours or that of the robot, will earn you 50 ECU.
An incorrect answer will earn you 0 ECU.

B. Receiving information:

At the end of the session, eight more periods will be drawn from the 48 periods in Part 2. Each period will have the same chance to be drawn.

For each period where the number Y drawn is less than or equal to X, the number of ECU you are willing to pay, Y ECU will be deducted from your initial endowment.

C. Final Compensation:

ECU will be converted into U.S. Dollars at a ratio of 100 ECU = $2.

The compensation for your performance and the compensation for your response to the questionnaires will be added to your initial $9.

Before you begin the second part, you will complete the following comprehension quiz. It will begin with questions about evaluating the news.

Once you are ready, please click OK to continue.

3. Comprehension questionnaire

Comprehension questionnaire

Before you begin the second part*, you will complete the following comprehension questionnaire. It will start with questions about information evaluation.*

Once you're ready, click "OK" to continue.

Information evaluation - Case N°1:

Consider the case where you move the slider to -70.

What will the submitted response be?

A. The response will be FALSE.'
B. The response will be 'TRUE.'

[If the response submitted is A.]: Correct: This is the right answer. If you move the slider to -70, the submitted response will be 'FALSE.'

[If the response submitted is B.]: Incorrect: This is not the right answer. If you move the slider to -70, the submitted response will be 'FALSE.'

Information evaluation - Case N°1:

Consider the case where you think the information is false and move the slider to -70. If the randomly selected robot has an accuracy level of 50:

What will the submitted evaluation be?

A. The evaluation submitted will be yours.
B. The evaluation submitted will the robot’s.

[If the response submitted is A.]: Correct: This is the right answer. If you move the slider to -70 and the randomly selected robot has an accuracy level of 50, the submitted evaluation will be yours.

[If the response submitted is B.]: Incorrect: This is not the right answer. If you move the slider to -70 and the randomly selected robot has an accuracy level of 50, the submitted evaluation will be yours.

Information evaluation - Case N°1:

Consider the case where you think an information is false. If your accuracy threshold is lower than that of the robot, and the randomly selected robot has an accuracy level of 50:

What are the chances out of 100 that the robot will submit the correct response?

[Slider response]

[If the slider response is 50]: Correct: This is the right answer. If your accuracy threshold is lower than that of the robot and the randomly selected robot has an accuracy level of 50, there is a 50% chance the robot will submit the correct response.

[If the slider response is not 50]: Incorrect: This is not the right answer. If your accuracy threshold is lower than that of the robot and the randomly selected robot has an accuracy level of 50, there is a 50% chance the robot will submit the correct response.

Information evaluation - Case N°2:

Consider the case where you move the slider to 50.

What will the submitted response be?

A. The response will be FALSE.'
B. The response will be 'TRUE.'

[If the response submitted is A.]: Incorrect: This is not the right answer. If you move the slider to 50, the submitted response will be 'TRUE.'

[If the response submitted is B.]: Correct: This is the right answer. If you move the slider to 50, the submitted response will be 'TRUE.'

Information evaluation - Case N°2:

Consider the case where you think an information is true and move the slider to the value 50. If the randomly selected robot has an accuracy level of 75:

What will the submitted evaluation be?

A. The evaluation submitted will be yours.
B. The evaluation submitted will the robot’s.

[If the response submitted is A.]: Incorrect: This is not the right answer. If you move the slider to 50 and the randomly selected robot has an accuracy level of 75, the submitted evaluation will be the robot's.

[If the response submitted is B.]: Correct: This is the right answer. If you move the slider to 50 and the randomly selected robot has an accuracy level of 75, the submitted evaluation will be the robot's.

Information evaluation - Case N°2:

Consider the case where you think an information is true. If your accuracy threshold is lower than that of the robot, and the randomly selected robot has an accuracy level of 75:

What are the chances out of 100 that the robot will submit the correct response?
[Slider response]

[If the slider response is 75]: Correct: This is the right answer. If your accuracy threshold is lower than that of the robot and the randomly selected robot has an accuracy level of 75, there is a 75% chance the robot will submit the correct response.

If the slider response is not 75]: Incorrect: This is not the right answer. If your accuracy threshold is lower than that of the robot and the randomly selected robot has an accuracy level of 75, there is a 75% chance the robot will submit the correct response.

Information evaluation - Case N°3:

Consider the case where you move the slider to -40.

What will the submitted response be?

A. The response will be FALSE.'
B. The response will be 'TRUE.'

[If the response submitted is A.]: Incorrect: This is not the right answer. If you move the slider to -40, the submitted response will be 'FALSE.'

[If the response submitted is B.]: Correct: This is the right answer. If you move the slider to -40, the submitted response will be 'FALSE.'

Information evaluation - Case N°3:

Consider the case where you think an information is false and move the slider to the value -40. If the randomly selected robot has an accuracy level of 62:

What will the submitted evaluation be?

A. The evaluation submitted will be yours.
B. The evaluation submitted will the robot's.

[If the response submitted is A.]: Incorrect: This is not the right answer. If you move the slider to -40 and the randomly selected robot has an accuracy level of 62, the submitted evaluation will be the robot's.

[If the response submitted is B.]: Correct: This is the right answer. If you move the slider to -40 and the randomly selected robot has an accuracy level of 62, the submitted evaluation will be the robot's.

Information evaluation - Case N°3:

Consider the case where you think an information is false. If your accuracy threshold is lower than that of the robot, and the randomly selected robot has an accuracy level of 62:

What are the chances out of 100 that the robot will submit the correct response?
[Slider response]

[If the slider response is 62]: Correct: This is the right answer. If your accuracy threshold is lower than that of the robot and the randomly selected robot has an accuracy level of 62, there is a 62% chance the robot will submit the correct response.

If the slider response is not 62]: Incorrect: This is not the right answer. If your accuracy threshold is lower than that of the robot and the randomly selected robot has an accuracy level of 62, there is a 62% chance the robot will submit the correct response.

Information reception - Case N°1:

Consider the case where this period is randomly selected at the end of the task to implement your decision:

You chose to receive additional information and stated that you are willing to pay 20 UME to receive more information.

If the program randomly selects the number 23, will you receive the additional information at the end of the session?

A. Yes.
B. No.

[If the response submitted is A.]: Incorrect: This is not the right answer. If a period is randomly selected at the end of the session and you stated you are willing to pay 20 UME for additional information, and the program selected the number 23, you will not receive the additional information at the end of the session.

If the response submitted is B.]: Correct: This is the right answer. If a period is randomly selected at the end of the session and you stated you are willing to pay 20 UME for additional information, and the program selected the number 23, you will not receive the additional information at the end of the session.

Information reception - Case N°1:

Consider the case where this period is randomly selected at the end of the task to implement your decision:

You stated you are willing to pay 20 UME to receive more information.

If the program randomly selects the number 23, will your initial allocation decrease for choosing to receive additional information?

A. Yes.
B. No.

[If the response submitted is A.]: Incorrect: This is not the right answer. If a period is randomly selected at the end of the session and you stated you are willing to pay 20 UME for additional information, and the program selected the number 23, your initial allocation will not decrease.

If the response submitted is B.]: Correct: This is the right answer. If a period is randomly selected at the end of the session and you stated you are willing to pay 20 UME for additional information, and the program selected the number 23, your initial allocation will not decrease.

Information reception - Case N°2:

Consider the case where this period is randomly selected at the end of the task to implement your decision:

You chose not to receive additional information and stated that you are willing to pay 10 UME not to receive more information.

If the program randomly selects the number 8, will you receive the additional information at the end of the session?

A. Yes.
B. No.

[If the response submitted is A.]: Incorrect: This is not the right answer. If a period is randomly selected at the end of the session and you stated you are willing to pay 10 UME not to receive additional

information, and the program selected the number 8, you will not receive the additional information at the end of the session.

If the response submitted is B.]: Correct: This is the right answer. If a period is randomly selected at the end of the session and you stated you are willing to pay 10 UME not to receive additional information, and the program selected the number 8, you will not receive the additional information at the end of the session.

Information reception - Case N°2:

Consider the case where this period is randomly selected at the end of the task to implement your decision:

You stated you are willing to pay 10 UME to receive more information.

If the program randomly selects the number 8, will your initial allocation decrease for choosing to receive additional information?

A. Yes.
B. No.

[If the response submitted is A.]: Correct: This is the right answer. If a period is randomly selected at the end of the session and you stated you are willing to pay 10 UME not to receive additional information, and the program selected the number 8, your initial allocation will decrease.

If the response submitted is B.]: Incorrect: This is not the right answer. If a period is randomly selected at the end of the session and you stated you are willing to pay 10 UME not to receive additional information, and the program selected the number 8, your initial allocation will decrease.

Information reception - Case N°2:

Consider the case where this period is randomly selected at the end of the task to implement your decision:

You stated you are willing to pay 10 UME to receive more information.

If the program randomly selects the number 8, by how many UME will your initial allocation decrease?
[Slider response]

[If the response submitted is 8.]: Correct: This is the right answer. If a period is randomly selected at the end of the session and you stated you are willing to pay 10 UME not to receive additional information, and the program selected the number 8, your initial allocation will decrease by 8 UME.

If the response submitted is not 8.]: Incorrect: This is not the right answer. If a period is randomly selected at the end of the session and you stated you are willing to pay 10 UME not to receive additional information, and the program selected the number 8, your initial allocation will decrease by 8 UME.

Information reception - Case N°3:

Consider the case where this period is randomly selected at the end of the task to implement your decision:

You chose to receive additional information and stated that you are willing to pay 18 UME to receive more information.

If the program randomly selects the number 17, will you receive the additional information at the end of the session?

C. Yes.
D. No.

[If the response submitted is A.]: Correct: This is the right answer. If a period is randomly selected at the end of the session and you stated you are willing to pay 18 UME for additional information, and the program selected the number 17, you will receive the additional information at the end of the session.

If the response submitted is B.]: Incorrect: This is not the right answer. If a period is randomly selected at the end of the session and you stated you are willing to pay 18 UME for additional information, and the program selected the number 17, you will receive the additional information at the end of the session.

Information reception - Case N°3:

Consider the case where this period is randomly selected at the end of the task to implement your decision:

You stated you are willing to pay 18 UME to receive more information.

If the program randomly selects the number 8, will your initial allocation decrease for choosing to receive additional information?

C. Yes.
D. No.

[If the response submitted is A.]: Correct: This is the right answer. If a period is randomly selected at the end of the session and you stated you are willing to pay 18 UME for additional information, and the program selected the number 17, your initial allocation will decrease.

If the response submitted is B.]: Incorrect: This is not the right answer. If a period is randomly selected at the end of the session and you stated you are willing to pay 18 UME for additional information, and the program selected the number 17, your initial allocation will decrease.

Information reception - Case N°3:

Consider the case where this period is randomly selected at the end of the task to implement your decision:

You stated you are willing to pay 18 UME to receive more information.

If the program randomly selects the number 17, by how many UME will your initial allocation decrease?

[Slider response]

[If the response submitted is 17.]: Correct: This is the right answer. If a period is randomly selected at the end of the session and you stated you are willing to pay 18 UME for additional information, and the program selected the number 17, your initial allocation will decrease by 17 UME.

If the response submitted is not 17.]: Incorrect: This is not the right answer. If a period is randomly selected at the end of the session and you stated you are willing to pay 18 UME for additional information, and the program selected the number 17, your initial allocation will decrease by 17 UME.

The comprehension questionnaire is now complete.
You will now begin training to familiarize yourself with the second part.
Your answers here will not be counted.
Once this training is complete, the task will begin.

III. Supplementary Methods III: Presentation of the organizations (translated from French)

1. Democracy-related organizations

France FREXIT

France FREXIT is a private and independent initiative, created in March 2018 and aiming at informing, gathering, exchanging, proposing on the theme of Frexit. In all legality, without any violence, and in the respect of the Institutions. Taking advantage of the historical opportunity of the Frexit, FRANCE FREXIT proposes the complete reform of the Republic and the French State by a great FRENCH NATIONAL COORDINATION, based on a new formula of Power, including a truly democratic organization taking the best of the Republic and leaving the least good, and using modern means of communication: votes, electronic votes, draws, as well as some monarchical aspects, mainly in terms of spirituality and transmission of universal and traditional values of France. FRANCE FREXIT is politically opposed to the euro-extremist and euro-identitarian parties such as LaREM, LR, MODEM, EELV, PS, UDI, Parti Radical. FRANCE FREXIT does not support the euro-alternative parties and other decoy parties such as the RN, the LFI or DLF, the NPA, LO and some other euro-compatible parties.

Parti Libertarien

Our observation is the same as the majority of French people: the weight of the state and its scope of action are constantly increasing, hindering our freedoms more and more, with the catastrophic results that everyone can see. We are also facing an extremely worrying legislative inflation, the accumulation of standards, regulations and laws make the system incomprehensible for the majority of French people and impracticable for entrepreneurs. Only large groups and multinationals benefit from this complexity and can expand without real competition. These regulations paralyze any personal initiative, block the social elevator and increase inequalities. Beyond the dramatic consequences of such an intrusion of the state in the life of individuals, the latter, by wanting to regulate every aspect of our daily life, goes beyond its prerogatives and violates our fundamental right to manage our life as we see fit. We are libertarians and we consider that it is up to free individuals to write their own history.

Le Mouvement Européen – France

The European Movement - France has been mobilizing since 1950, across all generations, to bring to life a pluralist public debate on Europe. It deploys its activities around pedagogy, the organization of debate between citizens and the formulation of proposals to build Europe. It gathers thousands of volunteers gathered in more than 50 local sections, about twenty member organizations as well as a college of qualified personalities. Heir to the spirit of the founding fathers of Europe, the Movement is the first actor of civil society in France on European issues. It also mobilizes through its youth branch, the Young Europeans - France, which has 26 local groups throughout the territory. At the European level, the European Movement is also a member of the European Movement - International, a network of 39 organizations that make our commitment resonate throughout the continent. The European Movement-France is recognized as an association of general interest since July 22, 2016 and is also approved as an "educational association complementary to public education".

Fondation Robert Schuman

Created in 1991 and recognized as a public utility, the Robert Schuman Foundation works in favour of European construction. As a reference research center the Foundation develops studies on the European Union and its policies and promotes their content in France, Europe and abroad. The Foundation is an open and multinational network. Its main mission is to keep alive the spirit and inspiration of one of the "Fathers of Europe", Robert Schuman, and to promote European values and ideals both within and beyond the borders of the Union. The Foundation produces numerous studies on European policies which constitute a valuable source of information for all those who want to understand European issues and challenges. Its independence allows it to deal with all current issues in an in-depth and objective manner. Its studies and analyses provide European decision-makers with information, arguments and food for thought that are appreciated for their usefulness and scientific quality. It multiplies initiatives in the field to advance the European democratic model.

2. Ecology-related organizations

Greenpeace

Since its creation some 50 years ago, Greenpeace has acted on land and sea according to the principles of non-violence to protect the environment and promote peace. Today, we remain faithful to this mission, as well as to our total financial and ideological independence. Climate change, growing inequality, social injustice, migration and armed conflict... All the major challenges of our time, to which we must urgently respond, are intimately linked - as are the power structures that create them and the mentalities that accommodate them. This is why it is necessary to transform them together. Greenpeace is present in 55 countries, on all continents and oceans, through its 28 national and regional offices and its three boats. It has more than three million members and over 36,000 volunteers worldwide. We place citizen power at the heart of our campaigns by giving resonance to the work of all those who share our vision, our hopes and our conviction that we need profound transformations in our societies.

World Wild Fund for Nature

WWF is one of the world's leading independent environmental organizations. WWF works to stop the degradation of the planet's natural environment and to build a future in which humans live in harmony with nature, conserving the world's biological diversity, ensuring the sustainable use of renewable natural resources, and promoting the reduction of pollution and waste. Since 1973, WWF France has been carrying out concrete actions to safeguard natural environments and their species, promote sustainable lifestyles, train decision-makers, support companies in reducing their ecological footprint, and educate young people. WWF France, a public utility foundation, works for a living planet from Paris, Marseille, the Alps, Guyana and New Caledonia. WWF is committed to action based on dialogue and respect for others, and adopts a global approach that takes into account the interdependence between the state of the planet and human development.

Groupe d'experts non-gouvernemental sur l'évolution du climat (NIPCC)

The Non-Governmental Panel on Climate Change (NIPCC) is an international group of non-governmental scientists and academics who have come together to present a comprehensive, reliable and realistic assessment of the science and economics of global warming. Because it is not a government agency, and because its members are not predisposed to believe that climate change is caused by human greenhouse gas emissions, the NIPCC is able to offer an independent "second opinion" to the evidence reviewed - or not reviewed - by the Intergovernmental Panel on Climate Change (IPCC) on the issue of global warming. Since its founding in 2008, the NIPCC has been producing publications and reports for public policy. These reports aim, for example, to show that the impact of human-induced global warming is benign and could be beneficial to humanity and the natural world; that the evidence for rising sea levels is unreliable; or that there is no scientific consensus in the climate change debate.

Association des climato-réalistes

Appeared in France in 2015, climatorealism sees the climate as an object of science and not ideology. Climate change is multiple and poorly understood, so there is no evidence that our way of life would cause "climate disruption." To say so is not selfishness, denial or anti-environmentalism, but realism. We need to think about how best to use our resources, and put our efforts where they really matter. The purpose of the association of climato-realists is to promote an open and free debate on the evolution of the climate and the societal and environmental issues related to it, by encouraging the expression of rigorous and well-founded opinions in all its forms. The association aims to make citizens aware of the stakes of climate and energy policies conducted in the name of the fight against global warming. The association is apolitical and totally free in the expression of its ideas. It strives to disseminate reliable information gathered from serious sources.

3. Social justice – related organizations

SOS Méditerranée

SOS Méditerranée is a European civil sea rescue association, independent of any political party and any religion, created in 2015 and made up of citizens mobilized to face the humanitarian emergency in the Mediterranean. SOS Méditerranée is based on the respect of man and his dignity, whatever his nationality, origin, social, religious, political or ethnic affiliation. The association's vocation is to provide assistance to any person in distress at sea who is within the scope of its action, without any discrimination. The persons concerned are men, women or children, migrants or refugees, who are in danger of death when crossing the Mediterranean Sea. The association also aims to ensure the protection of the survivors until their arrival in a safe port and to bear witness to the situation in the central Mediterranean. The association is financed by private donations and public grants. The funds collected are allocated to the rental of the boat, daily maintenance and rescue costs.

FEMEN

FEMEN is an international movement of feminist political activists with bare torsos, painted with slogans, and heads crowned with flowers. Our slogans are short and punchy; our chests are our banners. From the militant necessity is born the accomplishment of powerful and provocative but always non-violent actions. The movement was born in 2008 in Kiev, Ukraine. Since 2010, the activists are politicizing and using their breasts as a support for their demands. With our provocative and resounding actions, we target the multiple manifestations of the patriarchal order: dictatorships, sex industry and religions. We are a female revenge against the sclerotic patriarchal culture. We are an expression of freedom and pluralism. Our ambition is to change mentalities and the public image of women by exposing our strength, our courage and our convictions. It is by developing our political action that we will succeed in changing even our most intimate reality.

Génération Identitaire

Génération Identitaire is a political youth movement that brings together boys and girls across Europe. It was founded in September 2012. We call on young people to raise their heads: in the face of scum, in the face of those who want to control our lives and our thoughts, in the face of the standardization of peoples and cultures, in the face of the tidal wave of massive immigration, in the face of a school that hides the history of our people from us to prevent us from loving it, in the face of a so-called living together that turns into a nightmare... Génération Identitaire is the front line of resistance. Aware of the challenges we face, we do not refuse any battle. Proud of our heritage and confident in our destiny, we have only one watchword: we will not back down! We are the sacrificed generation, but not the lost generation. For we are going to war against all those who want to tear us away from our roots and make us forget who we are. Our ideal is reconquest, and we will carry it out to the end. Génération Identitaire is the barricade on which the youth in struggle for its identity stands.

La Manif Pour Tous

La Manif Pour Tous defends marriage and filiation in coherence with the sexual reality of humanity, whose consequence is both the difference and the complementarity of the sexes, which is essential to conceive a child and to assume the difference between father and mother, fatherhood and motherhood. Our goal is the respect of the superior interest and the elementary needs of the child, today threatened by the societal reforms inspired by the gender ideology. La Manif Pour Tous takes a pragmatic approach whose objective is to promote the well-being and the future of the child, the adult and society as a whole, what is commonly called the general interest. It acts for present and future generations. Finally, La Manif Pour Tous reminds us that the family is the crossroads of the difference between the sexes and the difference between generations. Only this context meets the essential needs of the child to come into the world, to know its personal identity, to enter little by little in relations with the others, to insert itself in the group then in the society, to contribute to the social peace.

IV. Supplementary Methods IV: Post-task questionnaires (translated from French)

1. Epistemic Curiosity questionnaire

Below are several statements that people use to describe themselves. Please read each statement and then select the appropriate response, using the scale below to indicate how you feel about these statements. There are no right or wrong answers. Please do not spend too much time on each statement but give the answer that describes how you generally feel.

1 = Almost Never 2 = Sometimes 3 = Often 4 = Almost Always

1. I enjoy exploring new ideas.
2. Difficult conceptual problems can keep me awake all night thinking about solutions.
3. I enjoy learning about subjects that are unfamiliar to me.
4. I can spend hours on a single problem because I just can't rest without knowing the answer.
5. I find it fascinating to learn new information.
6. I feel frustrated if I can't figure out the solution to a problem, so I work even harder to solve it.
7. When I learn something new, I would like to find out more about it.
8. I brood for a long time in an attempt to solve some fundamental problem.
9. I enjoy discussing abstract concepts.
10. I work like a fiend at problems that I feel must be solved.

2. Exposition to information

Now please read each statement below and select the appropriate answer(s). There are no right or wrong answers.

What information platforms do you use? Please check one box or more.

□Television

□ Radio

□ Newspapers and magazines

□ Internet

□ Social networks

□ Your friends

How often do you consult information sources?

□ Less than 1 time per week

□ 1 time per week

□ 2-3 times per week

□ 4-5 times per week

□ 1 time per day

□ 2-3 times per day

□ 4-5 times per day

□ More than 4-5 times per day

How many different news sources do you consult regularly?

3. Perceived percentage of fake news

Please answer each question below by dragging the slider (in the case of a default answer, please move the slider to activate it):

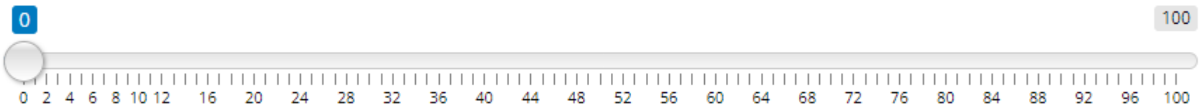

On social networks, in your opinion, what is the percentage of fake news when the information comes from a journalist?

On social networks, in your opinion, what is the percentage of fake news when the information from a politician?

On social networks, in your opinion, what is the percentage of fake news when the information comes from a doctor?

On social networks, in your opinion, what is the percentage of fake news when the information comes from a researcher?

On social networks, in your opinion, what is the percentage of fake news when the information comes from a social justice actor?

On social networks, in your opinion, what is the percentage of fake news when the information comes from an ecological actor?

On the internet, in general, in your opinion, what is the percentage of fake news?

4. Manipulation check

Regarding the experiment:

What was your strategy for choosing the number of chances out of 100 that the news was true or false?

What was your strategy for choosing what additional information to receive?

Did you think you would actually receive the additional information at the end of the experiment?

V. Supplementary Methods V: Sample size and data quality assessment

1. Sentiment analysis

We performed a sentiment analysis on the content of each stimulus. Sentiment analysis is a natural language processing technique used to determine whether the emotional valence of data is negative, neutral or positive. We used a multilingual XLM-roBERTa-base model sequence classifier based on Cardiff NLP Group sentiment classification model. The model was trained on ~198M tweets and fine-tuned for sentiment analysis on eight languages, including French. The analysis was performed with the Python package *transformers*. Out of the 96 stimuli, 90 had a probability score higher than 0.5 for the neutral dimension. In other words, 93.75% of the news were predominantly categorized as emotionally neutral.

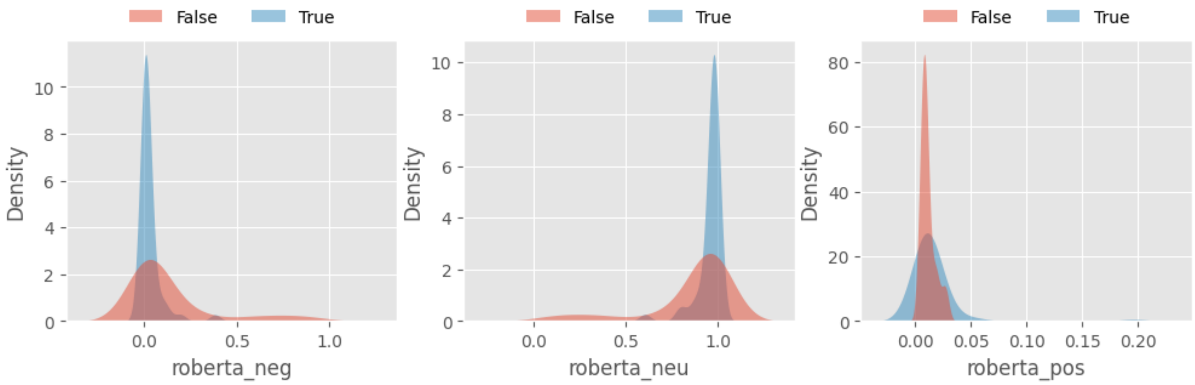

Supplementary Figure 1: The majority of stimuli were emotionally neutral. Density distributions of probability scores for the three emotional dimensions: negative, neutral and positive. The scores correspond to the softmax performed on the three classes with the sentiment-analysis pipeline from the Python package *transformers.*

2. Power simulation

We computed the sample size required to test the hypothesis of an effect of confidence, based on data from the first wave (N=79). We modelled the reception choices as a function of the confidence with repeated measures logistic mixed linear models.. To reduce computation time, we simplified the models by controlling only for the main effect of veracity judgment, and for the main and interaction effects of news veracity with news theme. The random structure only included subject random effects. The model yielded a fixed effect = -0.15 for the confidence variable. We set up a significance criterion of α = .05 and an estimated fixed effect = -0.72 before simulating the power for four levels of sample size (N = 80, N = 150, N = 200, N = 250). Investigating simulations, each sample size respectively yielded a power = .97 (CI: 91.48, 99.38); power = .99 (CI: 94.55, 99.97); power = .99 (CI: 96.38, 100); and power = .99 (CI: 96.38, 100).

3. Distributions of success in estimating veracity

We checked that the data collected in the two waves did not differ. No significant differences were detected between the first and second waves with respect to the average response time (RT) to estimate veracity (14.41 ± 8.44s; $ranksum$ p=.089). We investigated the probability to successfully estimate the veracity of news for both datasets and both groups with Bayesian beta-binomial models via RJAGS (Jeffreys priors: $\alpha = 0.5$, $\beta = 0.5$). Five Markov Chain Monte-Carlo (MCMC) simulations were run to approximate posterior distributions, comprising 12000 iterations, including 2000 warmup iterations, and a thinning rate of 5.

The delta of the beta-binomial posterior probabilities in the two datasets was equal to 0.002 (95% Credible Interval [-0.016, 0.021]). The delta of the two groups' posterior probabilities was equal to 0.025 (95% Credible Interval [0.008, 0.043]). Given the absence of statistically significant difference in the success probability between the two datasets and the 2.5% difference between groups, we pooled together data from the two waves and the two groups for the main analysis.

4. Distributions of demand for further information and related WTP

The average decision time was 1.56s (SD=1.12s) for the binary decision to receive or not further information and 2.84s (SD=0.88s) for the Willingness-to-Pay elicitation. The average response times in the second wave of data collection were significantly smaller for both reception choices (M=1.44s, SD=1.08s, $ranksum$ $p<0.0001$) and WTP (M=2.78s, SD=0.91s, $ranksum$ $p<0.05$). We investigated the probability to demand more information and the associated WTP with Bayesian beta-binomial models (Jeffreys priors: $\alpha = 0.5$, $\beta = 0.5$) and Bayesian normal distribution models (Jeffreys priors: $\mu = 0$, $\sigma = 1$ from half-Cauchy distribution) with RJAGS, respectively. We ran five Markov Chain Monte-Carlo (MCMC) simulations to approximate posterior distributions, with 12000 iterations including 2000 warmup, and a thinning rate of 5.

The delta of the two waves posterior probabilities of the demand for information was equal to 0.043 (95% Credible Interval [0.024, 0.062]). The delta of the two groups was equal to -0.043 (95% Credible Interval [-0.061, -0.026]). Participants in the two waves were each 4.3% more akin to choose to receive more information (odds-ratios = 1.19).

The delta of the two waves for the WTP was equal to 1.7 (95% Credible Interval [0.257, 3.069]) with Cohen's *d* of 0.311. The delta of the two groups for the WTP was equal to -0.154 (95% Credible Interval [-1.467, 1.136]) and the Cohen's *d* equal to -0.028. Although the difference in WTP was negligible between groups, the WTP of participants from the second wave (5.51 ECU) was 23.7% lesser than those from the first wave (7.18 ECU).

Investigating the WTP posterior samples for choices to receive extra information, we found a high delta for a difference between the two waves of data acquisition (delta = 2.58, 95% Credible Interval [1.107, 2.091]). Comparatively, the difference for choices not to receive between the two waves was absent (delta = 0.754, 95% Credible Interval [-0.683, 2.256]).

VI. Supplementary Methods VI: Behavioral analyses

1. Chance-level veracity estimation

We compared participants' performances in estimating veracity with a theoretical random distribution. A bootstrap Welch two-sample t-test between participants' average successes and a randomly generated binomial distribution (draws n=48, p=.05, size N=258, reps = 10000, confidence level =.95) showed that this success rate was not significantly different from chance (*p*=.068).

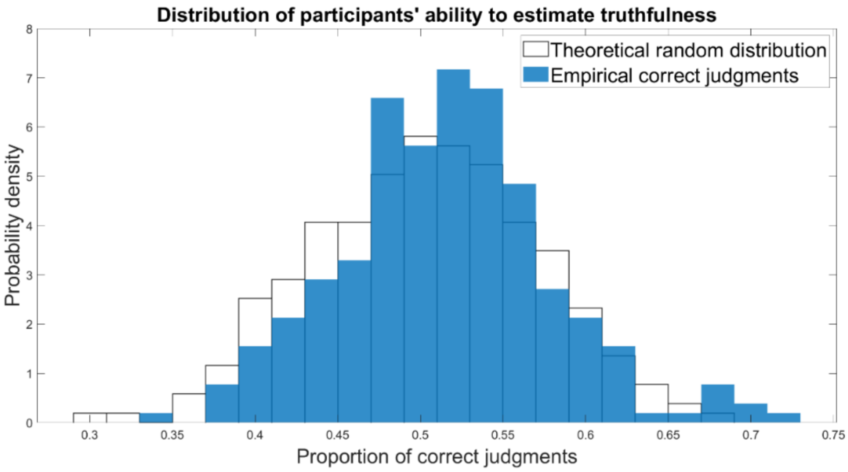

Supplementary Figure 2: Distribution of the proportion of participants' correct judgments against a theoretical random distribution (draws n=48, *p*=.05, size N=258)

We further computed the Bayes Factor of the hypothesis that *p*=.5 against the probability that *p* is not =.5. We defined a first logistic function with a prior for lambda = 0.5, a prior for *rscale* = 0.5 and iterated 10,000 times. Although this *rscale* is considered a medium value, it represents a tight distribution around the mean in our case. Given the observed success distribution at 51.6% (SD=6.7%), we also computed a logistic function with *rscale* = 1.5 for a wider distribution.

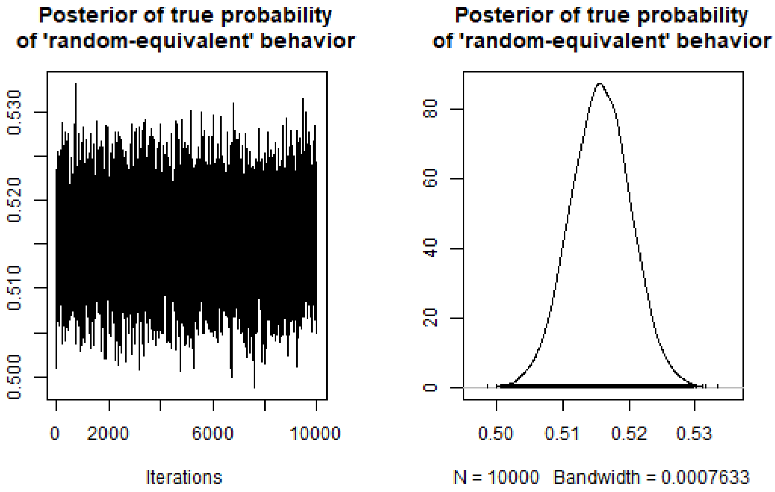

Supplementary Figure 3: Samples from the posterior distribution of the Bayesian logistic model (lambda = 0.5 and rscale = 0.5).

Supplementary Table 1: Summary of posterior distribution of the Bayesian logistic model (lambda = 0.5 and rscale = 0.5).

Parameter	Median	MAD	CI	CI_low	CI_high	pd	ROPE_CI	ROPE_low	ROPE_high	ROPE_Percentage	log_BF	BF	Prior_Distribution	Prior_Location	Prior_Scale
p	0.5156645	0.004494243	0.95	0.5066597	0.5243246	1	0.95	0.49	0.53	1	2.282283	9.799028			
Proportion													cauchy	0	0.5

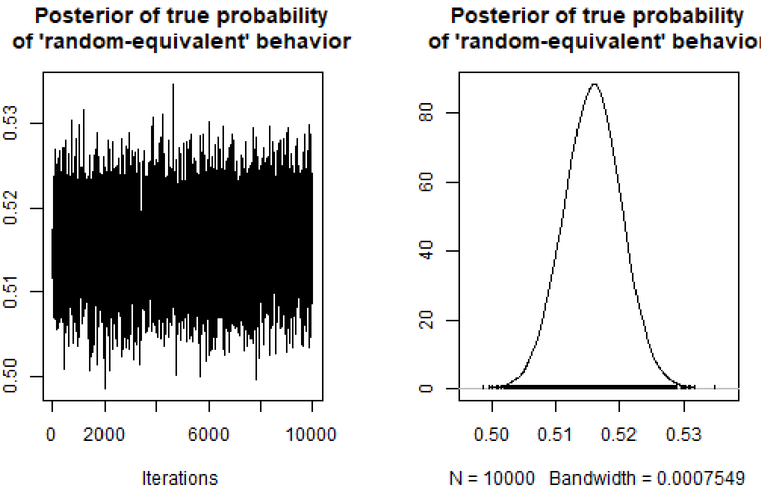

Supplementary Figure 4: Samples from the posterior distribution of the Bayesian logistic model (lambda = 0.5 and rscale = 1.5).

Supplementary Table 2: Summary of posterior distribution of the Bayesian logistic model (lambda = 0.5 and rscale = 1.5).

Parameter	Median	MAD	CI	CI_low	CI_high	pd	ROPE_CI	ROPE_low	ROPE_high	ROPE_Percentage	log_BF	BF	Prior_Distribution	Prior_Location	Prior_Scale
p	0.5154218	0.00448485	0.95	0.5068934	0.5243301	1	0.95	0.49	0.53	1	1.187445	3.278693			
Proportion													cauchy	0	1.5

2. Mixed Linear Models of veracity estimation

All behavioral hypotheses were tested with repeated measures Mixed Linear Models (MLMs). We modelled success in estimating veracity (correct or incorrect), veracity judgment (true or false), confidence (level per trial), demand for more information (choice to receive or not), and Willingness-To-Pay (ECUs amount per trial). The main independent variables we computed the MLMs with were news veracity (true or false), news theme (ecology, democracy or social justice), veracity judgment (true or false), confidence (continuous scale from 1 to 100), content imprecision (continuous scale from 1 to 10, as assessed by the independent raters; see Supplementary II) and content propensity to polarize (continuous scale from 1 to 10, as assessed by the independent raters; see Supplementary II). Due to the correlation between the imprecision and the polarization variables, we meaned centered both variables before orthogonalizing them with the Gram-Schmidt vector orthogonalization procedure.

Finally, we rescaled the two variables by dividing their respective centered values by their respective standard deviations. The confidence variable was rescaled as well. Age, Epistemic Curiosity, news content imprecision, confidence, and response times were rescaled and centered by dividing by the standard deviation and subtracting the mean.

To test the role of alignment/misalignment between subjects' beliefs and news-related concerns, we fitted a model for each theme, comprising the degree of adherence to relevant organizations (on a 1 to 100 continuous scale, normalized from participants' responses). For individual characteristics' effects, we constructed a model incorporating sociodemographics (age, sex, education, and epistemic curiosity). Cognitive reflection's role was tested using a model based on veracity estimation response times, while distrust in expert sources of information was evaluated using distrust scores (on a 1 to 100 continuous scale) from the final questionnaire. The test our null hypotheses, the random structure of our MLMs included random effects for participants. Including the random effects for the order of stimuli, waves and groups did not qualitatively change the results. Additionally, when behavioral independent variables were introduced as fixed effects, they were also included as random effects at the subject level. Post-hoc comparisons were conducted via simple slope comparisons with Bonferroni adjustments.

Supplementary Table 3: Performances (%) in correctly estimating veracity, classified by Theme and Veracity

Theme	Truthfulness	Mean	SD
Democracy	False	41.91	17.42
	True	55.38	19.93
Ecology	False	35.17	18.2
	True	69.28	17.05
Social justice	False	40.21	18.28
	True	67.44	17.43

Supplementary Table 4: News theme and news veracity as determinants of success in assessing the veracity of news

	Success			Success			Success		
Predictors	*Odds Ratios*	*CI*	*p*	*Odds Ratios*	*CI*	*p*	*Odds Ratios*	*CI*	*p*
(Intercept)	0.64	0.61 – 0.68	**<0.001**	0.54	0.50 – 0.59	**<0.001**	0.43	0.27 – 0.67	**<0.001**
Veracity: False	*Reference*			*Reference*			*Reference*		
Veracity: True	2.77	2.58 – 2.98	**<0.001**	4.16	3.65 – 4.73	**<0.001**	4.35	3.78 – 5.00	**<0.001**
Theme: Ecology	*Reference*			*Reference*			*Reference*		
Theme: Social Justice				1.24	1.09 – 1.41	**0.001**	1.24	1.08 – 1.42	**0.002**
Theme: Democracy				1.33	1.17 – 1.51	**<0.001**	1.37	1.19 – 1.56	**<0.001**
VeracityTrue:ThemeSocial Justice				0.74	0.62 – 0.89	**0.001**	0.73	0.60 – 0.89	**0.002**
VeracityTrue:ThemeDemocracy				0.41	0.35 – 0.50	**<0.001**	0.38	0.31 – 0.46	**<0.001**
Judgment: False	*Reference*			*Reference*			*Reference*		
Judgment: True							0.88	0.81 – 0.96	**0.003**
Confidence							1.03	0.99 – 1.08	0.128
Age							0.99	0.94 – 1.04	0.662
Sex: Female	*Reference*			*Reference*			*Reference*		
Sex: Male							1.06	0.97 – 1.15	0.177
Epistemic_curiosity							1.02	0.98 – 1.06	0.420
Education: Brevet	*Reference*			*Reference*			*Reference*		
Education: Bac							1.32	0.85 – 2.04	0.213
Education: Licence							1.24	0.77 – 1.99	0.376
Education: Master							1.30	0.83 – 2.03	0.249
**Random Effects**									
σ^2	3.29			3.29			3.29		
τ_{00}	0.00 $_{\text{Subject}}$			0.00 $_{\text{Subject}}$			0.01 $_{\text{Subject}}$		
τ_{11}							0.01 $_{\text{Subject.JudgmentTrue}}$		
							0.02 $_{\text{Subject.Confidence}}$		
ρ_{01}							-0.98		
							0.68		
N	258 $_{\text{Subject}}$			258 $_{\text{Subject}}$			228 $_{\text{Subject}}$		
Observations	12384			12384			10944		
Marginal R^2 / Conditional R^2	0.073 / NA			0.086 / NA			0.090 / NA		

We fitted mixed linear models (estimated using REML) to predict the success in estimating veracity with the news veracity (left) or its interaction with the news theme (middle). We investigated in the third model (right) what effect withstands the inclusion of control variables. Specifically, we included the veracity judgment, the confidence and the sociodemographics as control variables. The models included subjects, waves, groups and stimuli presentation order as random effects. Education: Brevet = Below High School degree, Bac = Final High School degree, Licence = Bachelor, Master = Master.

Supplementary Table 5: Proportion (%) of news judged as true, classified by Theme and Veracity

Theme	Truthfulness	Mean	SD
Democracy	False	56.15	19.04
	True	50.39	21.09
Ecology	False	61.00	18.24
	True	67.93	17.97
Social justice	False	57.46	17.85
	True	64.34	17.41

Supplementary Table 6: News theme and news veracity as determinants of the veracity judgment

	**Judgment**			**Judgment**			**Judgment**		
Predictors	*Odds Ratios*	*CI*	*p*	*Odds Ratios*	*CI*	*p*	*Odds Ratios*	*CI*	*p*
(Intercept)	1.41	1.32 – 1.50	**<0.001**	1.58	1.43 – 1.75	**<0.001**	2.30	1.18 – 4.52	**0.015**
Veracity: False	*Reference*			*Reference*			*Reference*		
Veracity: True	1.12	1.04 – 1.21	**0.002**	1.37	1.20 – 1.55	**<0.001**	1.41	1.23 – 1.63	**<0.001**
Theme: Ecology	*Reference*			*Reference*			*Reference*		
Theme: Social Justice				0.86	0.76 – 0.98	**0.019**	0.85	0.74 – 0.98	**0.023**
Theme: Democracy				0.81	0.72 – 0.92	**0.001**	0.79	0.69 – 0.91	**0.001**
VeracityTrue:ThemeSocial Justice				0.99	0.82 – 1.18	0.882	0.95	0.78 – 1.16	0.625
VeracityTrue:ThemeDemocracy				0.58	0.48 – 0.69	**<0.001**	0.54	0.45 – 0.66	**<0.001**
Confidence							1.03	0.96 – 1.11	0.371
Age							0.98	0.91 – 1.05	0.511
Sex: Female	*Reference*			*Reference*			*Reference*		
Sex: Male							1.05	0.93 – 1.19	0.426
Epistemic_curiosity							0.99	0.93 – 1.06	0.828
Education: Brevet	*Reference*			*Reference*			*Reference*		
Education: Bac							0.66	0.34 – 1.29	0.226
Education: Licence							0.64	0.31 – 1.32	0.227
Education: Master							0.74	0.38 – 1.47	0.393
**Random Effects**									
σ^2	3.29			3.29			3.29		
τ_{00}	0.12 $_{\text{Subject}}$			0.12 $_{\text{Subject}}$			0.12 $_{\text{Subject}}$		
τ_{11}							0.17 $_{\text{Subject.Confidence}}$		
ρ_{01}							0.45 $_{\text{Subject}}$		
ICC	0.03			0.04			0.08		
N	258 $_{\text{Subject}}$			258 $_{\text{Subject}}$			228 $_{\text{Subject}}$		
Observations	12384			12384			10944		
Marginal R^2 / Conditional R^2	0.001 / 0.035			0.017 / 0.052			0.022 / 0.100		

We fitted mixed linear models (estimated using REML) to predict the veracity judgment of news with the news veracity (left) or its interaction with the news theme (middle). We investigated in the third model (right) what effect withstands the inclusion of control variables. Specifically, we included the confidence and the sociodemographics as control variables. The models included subjects, waves, groups and stimuli presentation order as random effects. Education: Brevet = Below High School degree, Bac = Final High School degree, Licence = Bachelor, Master = Master.

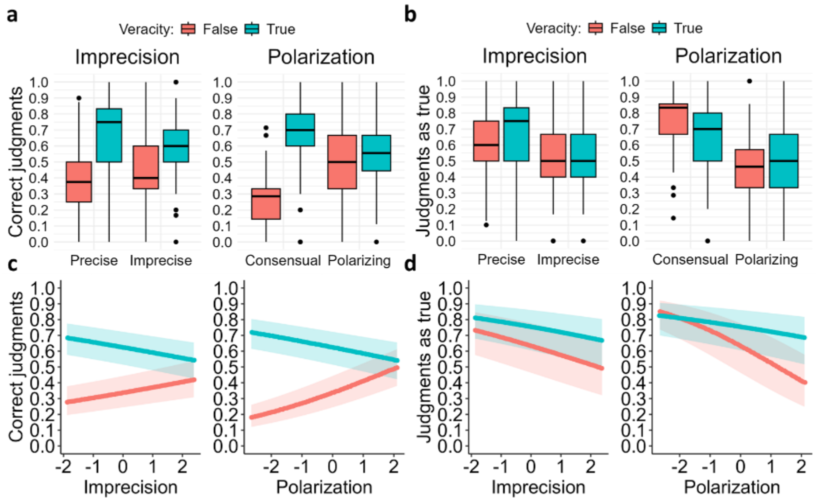

Supplementary Figure 5. News content imprecision and propensity to polarize serve as markers of falsity of the news. Plots of news content imprecision and news content propensity for polarization modulated by news veracity judgment on: **a)** the success in judging news veracity (*i.e.*, correctly assessing news veracity) and **b)** the likelihood of evaluating a news as true. Both effects are shown respectively in **c)** and **d)**, controlled for the effects of confidence, news themes modulated by news veracity and the random effects. **c)** Prediction success was more likely for true news when their content imprecision was at its minimum rather than maximum. It was also more likely for true news when their content polarization was at its *minimum*. **d)** The likelihood of assessing a news as true decreased as both imprecision and content polarization increased. Note: n=258.

Supplementary Table 7: Level of imprecision and level of polarization of information content as determinant of success in assessing the veracity of news, the veracity judgment and the confidence in the veracity judgment

	Success			Judgment			Confidence		
Predictors	*Odds Ratios*	*CI*	*p*	*Odds Ratios*	*CI*	*p*	*Estimates*	*CI*	*p*
(Intercept)	0.51	0.32 – 0.79	**0.003**	1.73	0.87 – 3.46	0.121	-0.43	-1.22 – 0.36	0.290
Veracity: False	*Reference*			*Reference*			*Reference*		
Veracity: True	3.28	2.82 – 3.80	**<0.001**	1.78	1.53 – 2.07	**<0.001**	0.04	-0.01 – 0.10	0.114
Imprecision	1.16	1.09 – 1.23	**<0.001**	0.78	0.74 – 0.83	**<0.001**	0.02	-0.00 – 0.04	0.098
Polarization	1.37	1.29 – 1.46	**<0.001**	0.64	0.60 – 0.68	**<0.001**	0.10	0.07 – 0.13	**<0.001**
Judgment: False	*Reference*			*Reference*			*Reference*		
Judgment: True	0.93	0.85 – 1.01	0.070				0.01	-0.02 – 0.05	0.375
VeracityTrue:Imprecision	0.75	0.69 – 0.81	**<0.001**	1.07	0.98 – 1.16	0.130			
VeracityTrue:Polarization	0.62	0.57 – 0.68	**<0.001**	1.33	1.22 – 1.46	**<0.001**			
JudgmentTrue:Imprecision							-0.05	-0.08 – -0.02	**0.002**
JudgmentTrue:Polarization							-0.15	-0.18 – -0.12	**<0.001**
Theme: Ecology	*Reference*			*Reference*			*Reference*		
Theme: Social Justice	0.96	0.83 – 1.10	0.541	1.25	1.08 – 1.45	**0.003**	0.09	0.04 – 0.14	**0.001**
Theme: Democracy	1.06	0.92 – 1.23	0.419	1.17	1.01 – 1.36	**0.040**	-0.16	-0.21 – -0.10	**<0.001**
VeracityTrue:ThemeSocial Justice	1.08	0.88 – 1.32	0.488	0.74	0.60 – 0.91	**0.005**	-0.09	-0.16 – -0.02	**0.018**
VeracityTrue:ThemeDemocracy	0.60	0.49 – 0.75	**<0.001**	0.45	0.36 – 0.56	**<0.001**	-0.00	-0.08 – 0.07	0.956
Confidence	1.05	1.00 – 1.09	**0.041**	1.02	0.95 – 1.10	0.519			
Epistemic_curiosity	1.02	0.98 – 1.06	0.386	0.99	0.93 – 1.06	0.847	0.05	-0.02 – 0.12	0.181
Age	0.99	0.94 – 1.04	0.660	0.97	0.91 – 1.05	0.470	-0.08	-0.16 – 0.00	0.062
Theme: Democracy	1.06	0.92 – 1.23	0.419	1.17	1.01 – 1.36	**0.040**	-0.16	-0.21 – -0.10	**<0.001**
Sex: Female	*Reference*			*Reference*			*Reference*		
Judgment: True	0.93	0.85 – 1.01	0.070				0.01	-0.02 – 0.05	0.375
Sex: Male	1.05	0.97 – 1.14	0.227	1.05	0.93 – 1.19	0.445	0.33	0.18 – 0.48	**<0.001**
Education: Brevet	*Reference*			*Reference*			*Reference*		
Education: Bac	1.33	0.86 – 2.06	0.194	0.65	0.33 – 1.30	0.222	0.21	-0.58 – 1.00	0.597
Education: Licence	1.26	0.79 – 2.03	0.336	0.63	0.30 – 1.31	0.214	-0.02	-0.89 – 0.84	0.956
**Random Effects**									
σ^2	3.29			3.29			0.66		
τ_{00}	0.00 Subject			0.13 Subject			0.30 Subject		
τ_{11}	0.01 Subject.Confidence			0.17 Subject.Confidence					
ρ_{01}	-0.98 Subject			0.42 Subject					
ICC	0.00			0.08			0.31		
N	228 Subject			228 Subject			228 Subject		
Observations	10944			10944			10944		
Marginal R^2 / Conditional R^2	0.109 / 0.113			0.057 / 0.135			0.054 / 0.350		

We fitted mixed linear models (estimated using REML) to predict the success in estimating veracity (left) and the veracity judgment (left) with the news veracity and its interaction with the level of imprecision of information content. We included as control variables the veracity judgment (left), the news themes, the confidence and the sociodemographics. The models included subjects, waves, groups and stimuli

presentation order as random effects. Education: Brevet = Below High School degree, Bac = Final High School degree, Licence = Bachelor, Master = Master.

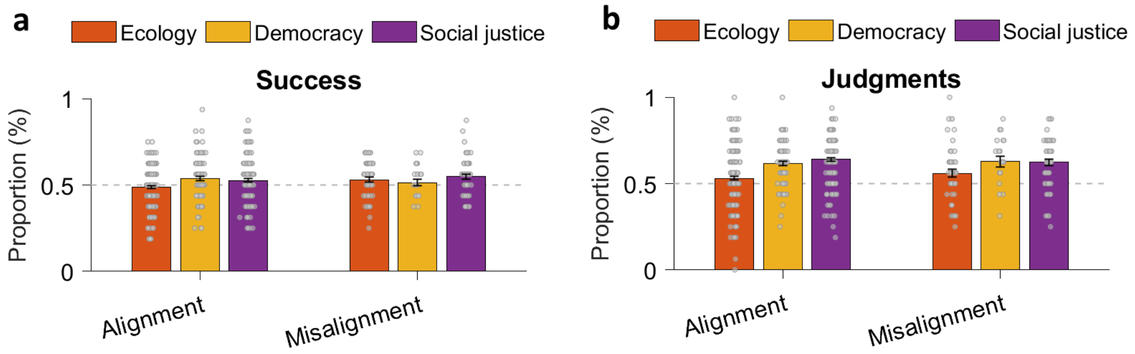

Supplementary Figure 6: Adhesion of organizations aligned or misaligned with concerns related to the themes had little effect on the proportion of a) successful trials and b) judgments of news as true. For each theme, we separated organizations in two subgroups with opposite views on the theme. Were considered WWF and Greenpeace as aligned with concerns related to ecology, Femen and SOS Méditerranée as aligned with those related to social justice, Fondation Robert Schuman and Le Mouvement Européen pour la Démocratie as aligned with those related to democracy. On the contrary, NIPCC and Association des Climato-Réalistes were considered misaligned with concerns related to ecology, Génération Identitaire and La Manif pour Tous misaligned with those related to social justice, Frexit and Parti Libertarien as misaligned with concerns related to democracy. Participants were considered aligned/misaligned when the mean adhesion to aligned/misaligned organizations were lower than 65/100. The figure illustrates how the alignment has little impact on the news veracity judgments and capacity to discern true from false news. Note: n=258.

Supplementary Table 8: Alignment/misalignment of one's own beliefs with concerns related to the news as determinant of success in assessing the veracity of news

	Ecology_success			Democracy_success			SocJust_success		
Predictors	*Odds Ratios*	*CI*	*p*	*Odds Ratios*	*CI*	*p*	*Odds Ratios*	*CI*	*p*
(Intercept)	0.91	0.59 – 1.40	0.662	0.98	0.72 – 1.33	0.907	1.27	0.87 – 1.88	0.218
Climato_realistes	1.00	1.00 – 1.00	0.925						
Greenpeace	1.00	0.99 – 1.00	0.686						
NIPCC	1.00	1.00 – 1.00	0.549						
WWF	1.00	1.00 – 1.01	0.372						
Fondation_Robert_Schuman				1.00	1.00 – 1.01	0.760			
Frexit				1.00	1.00 – 1.00	0.840			
Mouvement_europeen_france				1.00	0.99 – 1.00	0.690			
Parti_libertarien				1.00	1.00 – 1.00	0.634			
Femen							1.00	1.00 – 1.01	0.151
Generation_identitaire							1.00	0.99 – 1.00	0.084
La_manif_pour_tous							1.00	1.00 – 1.00	0.557
Sos_mediterranee							1.00	1.00 – 1.00	0.805
**Random Effects**									
σ^2	3.29			3.29			3.29		
τ_{00}	0.00 $_{Subject}$			0.00 $_{Subject}$			0.00 $_{Subject}$		
N	258 $_{Subject}$			258 $_{Subject}$			258 $_{Subject}$		
Observations	4128			4128			4128		
Marginal R^2 / Conditional R^2	0.000 / NA			0.000 / NA			0.002 / NA		

We fitted mixed linear models (estimated using REML) to predict the success in estimating veracity with the alignment/misalignment of subjects' beliefs with concerns related to the news. We estimated a model for news related to ecology (left), a model for news related to democracy (middle) and a model for news related to social justice (right). We included for each model the degree of adherence to organizations related to the news theme. The models included subjects, waves, groups and stimuli presentation order as random effects.

Supplementary Table 9: Socio-demographics, response times and distrust toward expert sources of information as determinants of success in assessing the veracity of news

	Success			Success			Success		
Predictors	*Odds Ratios*	*CI*	*p*	*Odds Ratios*	*CI*	*p*	*Odds Ratios*	*CI*	*p*
(Intercept)	0.54	0.50 – 0.59	**<0.001**	0.80	0.53 – 1.20	0.272	1.09	0.95 – 1.26	0.212
Estimation_RT	1.05	1.01 – 1.10	**0.030**						
Veracity: False	*Reference*			*Reference*			*Reference*		
Veracity: True	4.16	3.66 – 4.74	**<0.001**						
Theme: Ecology	*Reference*			*Reference*			*Reference*		
Theme: Social Justice	1.24	1.09 – 1.41	**0.001**						
Theme: Democracy	1.33	1.17 – 1.51	**<0.001**						
VeracityTrue:ThemeSocial Justice	0.74	0.62 – 0.89	**0.001**						
VeracityTrue:ThemeDemocracy	0.41	0.34 – 0.49	**<0.001**						
Age				0.99	0.95 – 1.03	0.559			
Sex: Female	*Reference*			*Reference*			*Reference*		
Sex: Male				1.06	0.98 – 1.14	0.122			
Epistemic_curiosity				1.02	0.98 – 1.06	0.313			
Education: Brevet	*Reference*			*Reference*			*Reference*		
Education: Bac				1.31	0.87 – 1.97	0.189			
Education: Licence				1.23	0.79 – 1.92	0.361			
Education: Master				1.30	0.86 – 1.97	0.209			
Distrust_environmental_activists							1.00	1.00 – 1.00	0.167
Distrust_general							1.00	1.00 – 1.00	0.542
Distrust_journalists							1.00	1.00 – 1.00	0.543
Distrust_physicians							1.00	1.00 – 1.00	0.845
Distrust_politics							1.00	1.00 – 1.00	0.892
Distrust_researchers							1.00	1.00 – 1.00	**0.045**
Distrust_socjust_activists							1.00	1.00 – 1.00	0.830
**Random Effects**									
σ^2	3.29			3.29			3.29		
τ_{00}	0.00 Subject			0.00 Subject			0.00 Subject		
							0.01 Order		
							0.00 Group		
τ_{11}	0.01 Subject.Estimation_RT								
ρ_{01}									
ICC							0.00		
N	258 Subject			228 Subject			179 Subject		
							48 Order		
							2 Group		
Observations	12384			10944			8592		
Marginal R^2 / Conditional R^2	0.086 / NA			0.001 / NA			0.001 / 0.003		

We fitted mixed linear models (estimated using REML) to predict the success in estimating veracity with the response times (far left and middle left), the sociodemographics (middle right) and the distrust in various institutions (far right). We investigated whether the response times effect withstands the inclusion of control variables. Specifically, we included the confidence, the veracity judgment and its interaction with the news theme as control variables. The models included subjects, waves, groups and

stimuli presentation order as random effects. Education: Brevet = Below High School degree, Bac = Final High School degree, Licence = Bachelor, Master = Master. Distrust in researchers as sources of information on social media was the only variable of distrust to significantly improve success in assessing news veracity (p=0.045, odds-ratio = 1).

3. Comparison of Bayesian Mixed Linear Models of veracity estimation

We tested Bayesian hypotheses with separate Bayesian multilevel linear models for each dependent variable. We computed Bayesian beta-binomial Mixed Linear Models (MLM) with the package *brms* for R. Each model was computed with 4 MCMC chains, 4000 iterations per chain plus 1500 warmup iterations. We chose a thinning = 1 and a delta = .85. The default delta is .8 and maximum recommended delta is 1. Increasing the delta slows down the sampler but decreases the number of divergent transitions. We chose weakly informative priors for every model; with *mu*[0,1] and *sigma*[0,1], except for the *non-informative beta response* model that took α = 0.5, β = 0.5 as priors for the intercept. Weakly informative and non-informative priors specifically allow data dominance over priors.

The Bayesian models aligned with models formulated for null hypothesis significance testing. Each model included the variables of interest and a simplified random structure, consisting solely of subject random effects (intercepts), to save computation time. We tested a *non-informative beta response* model to model randomness, with priors for the intercept α = 0.5 and β = 0.5. We included a *subject random-effect* model comprising only subject random effects (intercepts). To assess alignment/misalignment of subjects' beliefs with news concerns, we identified the most relevant organization for each theme and constructed a *beliefs alignment* model using the degree of adherence to these organizations (continuous scale from 1 to 100, normalized from participants' responses). Cognitive reflection's role was examined through a *response time* model based on veracity estimation response times. A *veracity judgment* model assessing the tendency to declare news as true was estimated based on veracity judgment. We defined a *news content imprecision* model comprising the interaction effect of news imprecision and veracity and a *news content propensity to polarize* model including the interaction effect of polarization and veracity. Lastly, we assessed a *news content imprecision & polarization* model that combined the interaction effects from both individual models. We also tested the relationship between the accuracy in estimating veracity and the confidence in veracity judgment.

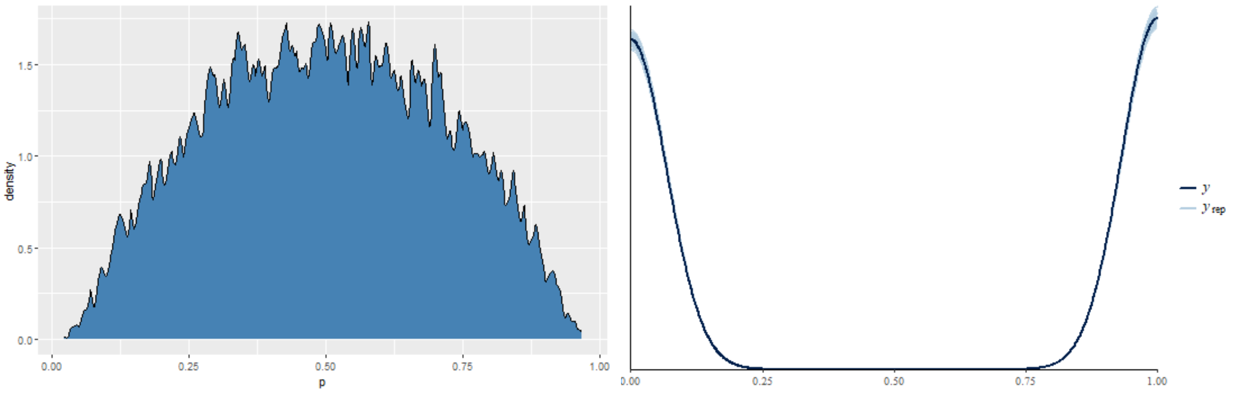

Supplementary Figure 7: Weakly-informative prior distribution for the Bayesian beta-binomial MLM of content precision, followed by the posterior prediction plotted against the observed data. The priors of winning model are widely distributed around p = .5 and the posterior closely map *y* the observed data.

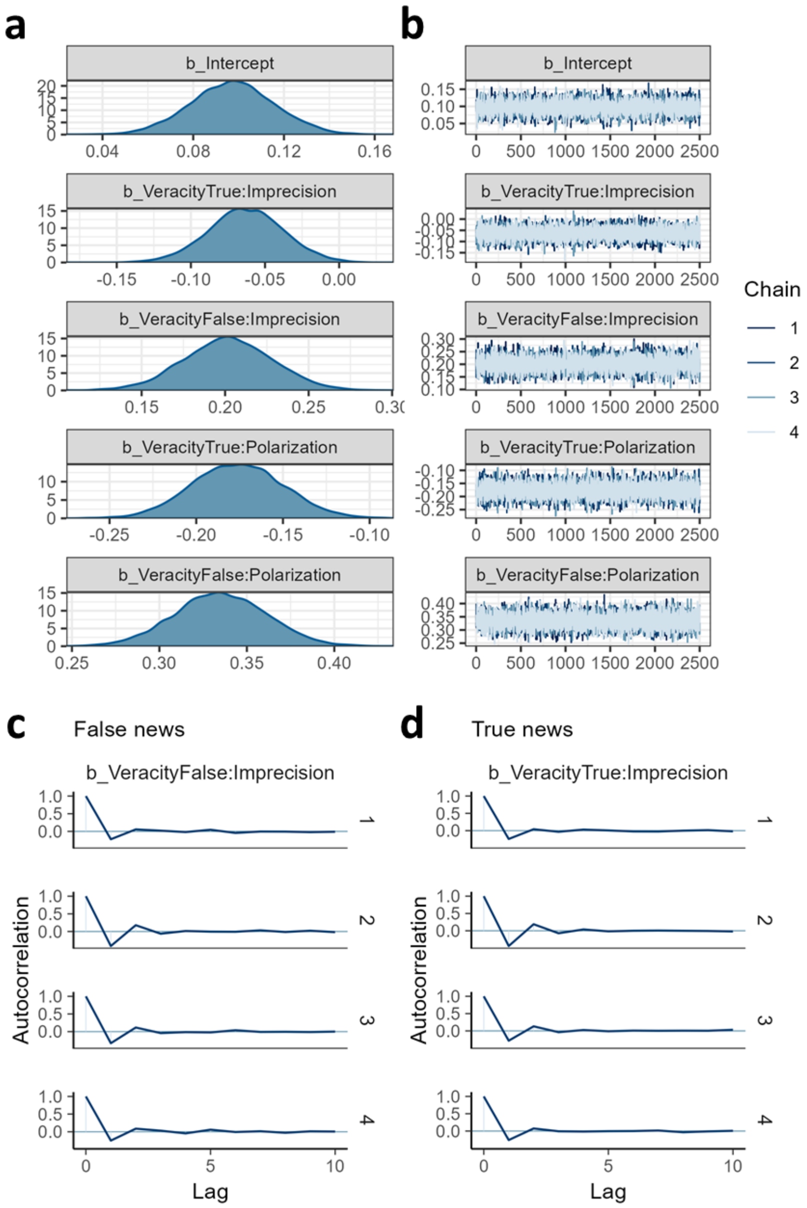

Supplementary Figure 8: Convergence plot followed by the autocorrelation plot, for the Bayesian beta-binomial MLM of content imprecision. **a)** The parameter values of the MLM for the effects of the imprecision and of the polarization on the success is positive when the news veracity is false, whereas it is negative when the news veracity is true. **b)** The 4 chains converged on the same parameter values and variations decrease over time. Moreover, the posterior samples for **c)** false news and **d)** true news seem uncorrelated after two lags. The autocorrelation plot is useful to check the posterior samples of each parameter. Positive autocorrelation means the chain tends to stay in the same area between iterations. Ideally, we want it to drop quickly to zero with increasing lag.

To check the interaction effect of news imprecision with news veracity, we also ran a second version of the model. This model took as fixed effects the main effects of news imprecision, news veracity, news theme, the interaction between the latter and the level of confidence. We kept the random structure as is and plotted the fixed effect of interest (Fig. S11 & S12).

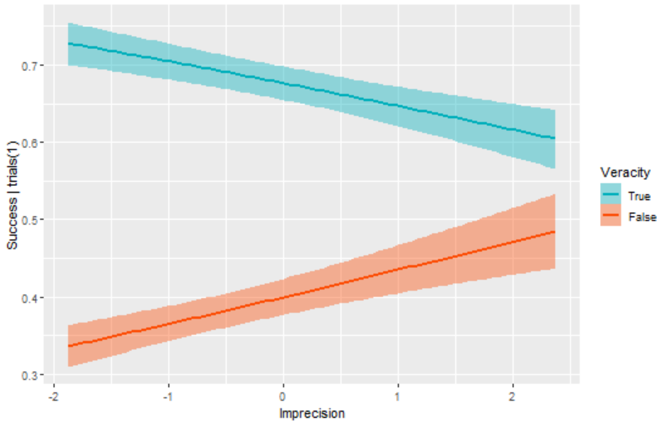

Supplementary Figure 9: Plot of the estimated effect of the interaction effect posterior samples. The estimated effects show that prediction success was more likely for true news when their content imprecision was at its minimum rather than maximum. It was more likely for false news when imprecision was at its maximum rather than minimum.

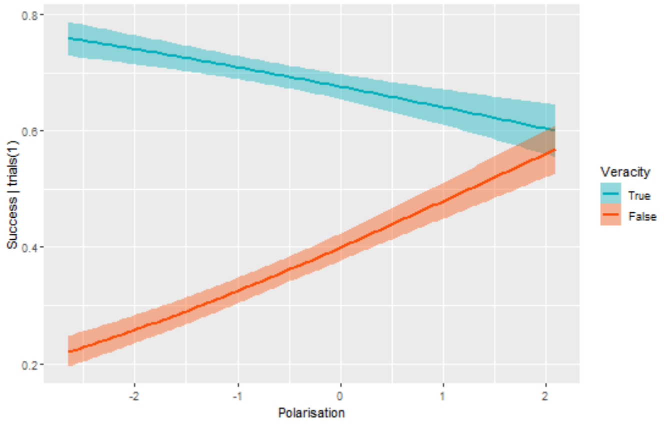

Supplementary Figure 10: Plot of the estimated effect of the interaction effect posterior samples. The estimated effects show that prediction success was more likely for true news when their content polarization was at its minimum rather than maximum. It was more likely for false news when polarization was at its maximum rather than minimum.

4. Mixed Linear Models of metacognitive abilities

Supplementary Table 10: Confidence in veracity estimation, classified by Theme and Veracity

Theme	Truthfulness	Mean	SD
Democracy	False	50.53	18.26
	True	51.66	17.98
Ecology	False	55.00	18.72
	True	55.83	18.21
Social justice	False	57.95	17.65
	True	56.20	17.86

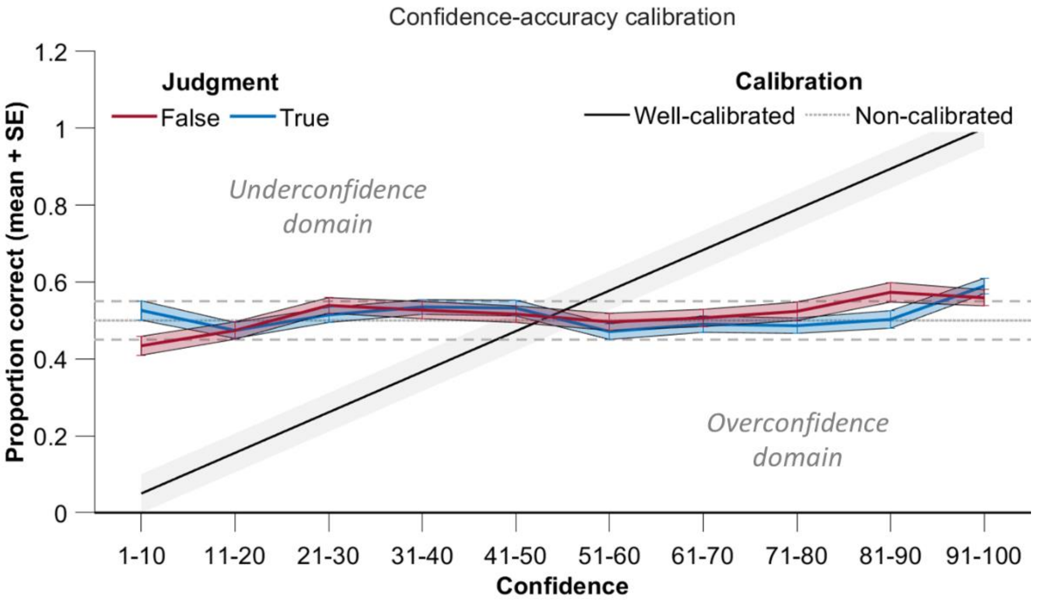

Supplementary Figure 11. Participants were not calibrated for estimating the probability of news veracity. The confidence-accuracy calibration plot displays the participants' accuracy in estimating probabilities that their judgment was correct, as a function of their confidence level. Well-calibrated estimated probabilities would intersect with confidence degrees in the grey area, meaning a 0-20 % confidence degree would lead to a 0-20 % accuracy in evaluating the news veracity. The plot shows that overall, the proportion of accurate veracity estimations neither increased nor decreased with confidence. Furthermore, the plot emphasizes that accuracy is higher for true news than false ones (the green curve always lies above the red one). Underconfidence dominates for true news whereas overconfidence dominates for false news. Note: n=258.

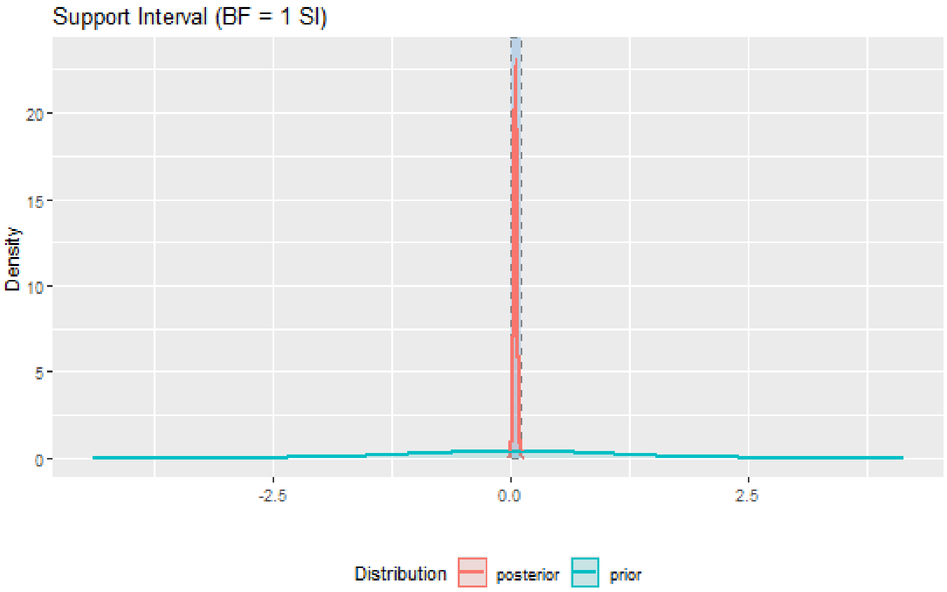

Supplementary Figure 12. Support interval for the effect of confidence on success in estimating news veracity. Bayesian analysis reveals a small positive coefficient for Confidence (β = 0.05, 95% CI [0.02, 0.09]), accounting for only 0.12% of the variance in Success (R^2 = 0.0012, 95% CI [0.0002, 0.003]). With 100% of the posterior distribution within the Region of Practical Equivalence (ROPE) [-0.1, 0.1] and an interval null Bayes Factor $BF_{01} = 1/1830$, there is stronger support for the null hypothesis. The support interval [0.00, 0.10], shown here, lies entirely within the ROPE.

Supplementary Table 11: Alignment/misalignment of subjects' beliefs with concerns related to the news as determinant of the confidence in the veracity assessment

	**Confidence**			**Confidence**			**Confidence**		
Predictors	*Estimates*	*CI*	*p*	*Estimates*	*CI*	*p*	*Estimates*	*CI*	*p*
(Intercept)	0.18	-0.36 – 0.73	0.512	-0.14	-0.52 – 0.23	0.451	0.38	-0.08 – 0.84	0.108
Climato_realistes	0.00	-0.00 – 0.00	0.884						
Greenpeace	-0.00	-0.01 – 0.00	0.425						
NIPCC	-0.00	-0.01 – 0.00	0.430						
WWF	0.00	-0.01 – 0.01	0.771						
Fondation_Robert_Schuman				0.00	-0.00 – 0.01	0.201			
Frexit				-0.00	-0.01 – 0.00	0.240			
Mouvement_europeen_france				-0.00	-0.01 – 0.00	0.423			
Parti_libertarien				0.00	-0.00 – 0.01	0.546			
Femen							-0.00	-0.01 – 0.00	0.552
Generation_identitaire							-0.00	-0.01 – 0.00	0.276
La_manif_pour_tous							-0.00	-0.00 – 0.00	0.663
Sos_mediterranee							-0.00	-0.01 – 0.00	0.717
**Random Effects**									
σ^2	0.65			0.63			0.69		
τ_{00}	0.36 $_{Subject}$			0.34 $_{Subject}$			0.32 $_{Subject}$		
ICC	0.36			0.35			0.31		
N	258 $_{Subject}$			258 $_{Subject}$			258 $_{Subject}$		
Observations	4128			4128			4128		
Marginal R^2 / Conditional R^2	0.002 / 0.359			0.005 / 0.352			0.003 / 0.317		

We fitted mixed linear models (estimated using REML) to predict the level of confidence in the veracity assessment with the alignment/misalignment of subjects' beliefs with concerns related to the news. We estimated a model for news related to ecology (left), a model for news related to democracy (middle) and a model for news related to social justice (right). We included for each model the degree of adherence to organizations related to the news theme. The models included subjects, waves, groups and stimuli presentation order as random effects.

Supplementary Table 12: Content imprecision as determinants of the degree of confidence in the veracity assessment

	Confidence			Confidence		
Predictors	*Estimates*	*CI*	*p*	*Estimates*	*CI*	*p*
(Intercept)	-0.03	-0.11 – 0.04	0.382	-0.43	-1.22 – 0.36	0.290
Judgment: False	*Reference*			*Reference*		
Judgment: True	0.03	0.00 – 0.06	**0.045**	0.01	-0.02 – 0.05	0.375
Imprecision	0.00	-0.02 – 0.03	0.699	0.02	-0.00 – 0.04	0.098
Polarization	0.09	0.07 – 0.11	**<0.001**	0.10	0.07 – 0.13	**<0.001**
JudgmentTrue:Imprecision	-0.06	-0.09 – -0.03	**<0.001**	-0.05	-0.08 – -0.02	**0.002**
JudgmentTrue:Polarization	-0.15	-0.18 – -0.12	**<0.001**	-0.15	-0.18 – -0.12	**<0.001**
Veracity: False	*Reference*			*Reference*		
Veracity: True				0.04	-0.01 – 0.10	0.114
Theme: Ecology	*Reference*			*Reference*		
Theme: Social Justice				0.09	0.04 – 0.14	**0.001**
Theme: Democracy				-0.16	-0.21 – -0.10	**<0.001**
VeracityTrue:ThemeSocial Justice				-0.09	-0.16 – -0.02	**0.018**
VeracityTrue:ThemeDemocracy				-0.00	-0.08 – 0.07	0.956
Estimation_RT				-0.05	-0.07 – -0.03	**<0.001**
Age				-0.08	-0.16 – 0.00	0.062
Sex: Female	*Reference*			*Reference*		
Sex: Male				0.33	0.18 – 0.48	**<0.001**
Epistemic_curiosity				0.05	-0.02 – 0.12	0.181
Education: Brevet	*Reference*			*Reference*		
Education: Bac				0.21	-0.58 – 1.00	0.597
Education: Licence				-0.02	-0.89 – 0.84	0.956
Education: Master				0.37	-0.44 – 1.17	0.371
**Random Effects**						
σ^2	0.67			0.66		
τ_{00}	0.32 $_{\text{Subject}}$			0.30 $_{\text{Subject}}$		
ICC	0.33			0.31		
N	258 $_{\text{Subject}}$			228 $_{\text{Subject}}$		
Observations	12384			10944		
Marginal R^2 / Conditional R^2	0.007 / 0.331			0.054 / 0.350		

We fitted mixed linear models (estimated using REML) to predict the subjects' degree of confidence in their veracity assessment with the news veracity and its interaction with the level of imprecision of information content (left). We investigated whether the response times effect withstands the inclusion of control variables. Specifically, we included the veracity judgment, the news themes, the response times and the sociodemographics (right). The models included subjects, waves, groups and stimuli presentation order as random effects. Education: Brevet = Below High School degree, Bac = Final High School degree, Licence = Bachelor, Master = Master.

5. Mixed Linear Models of demand for further information

Supplementary Table 13: Choices (%) to receive extra information, classified by Theme and Veracity

Theme	Truthfulness	Mean	SD
Democracy	False	41.48	34.59
	True	40.89	34.74
Ecology	False	43.70	34.63
	True	42.83	35.02
Social justice	False	42.64	34.47
	True	42.49	34.06

Supplementary Table 14: Willingness-To-Pay (from 0 to 25 ECU) to receive or not extra information, classified by Reception choice, Theme and Veracity

Reception	Theme	Truthfulness	Mean	SD
No reception	*Democracy*	False	5.72	6.04
		True	5.83	5.98
	Ecology	False	5.86	6.2
		True	5.95	6.23
	Social justice	False	5.71	6.04
		True	5.52	5.86
Reception	*Democracy*	False	7.37	5.47
		True	7.26	5.05
	Ecology	False	7.17	5.33
		True	7.45	5.5
	Social justice	False	7.6	5.57
		True	6.99	4.92

Supplementary Table 15: Degree of confidence in the veracity assessment as determinant of the decision to demand or not further information

	Reception			Reception		
Predictors	*Odds Ratios*	*CI*	*p*	*Odds Ratios*	*CI*	*p*
(Intercept)	0.41	0.29 – 0.58	**<0.001**	0.78	0.02 – 36.41	0.898
Confidence	0.59	0.52 – 0.67	**<0.001**	0.59	0.52 – 0.68	**<0.001**
Judgment: False	*Reference*			*Reference*		
Judgment: True	1.21	1.04 – 1.40	**0.012**	1.25	1.05 – 1.47	**0.010**
JudgmentTrue:Confidence	1.27	1.13 – 1.42	**<0.001**	1.29	1.14 – 1.46	**<0.001**
Veracity: False	*Reference*			*Reference*		
Veracity: True				0.87	0.73 – 1.04	0.130
Theme: Ecology	*Reference*			*Reference*		
Theme: Social Justice				0.91	0.76 – 1.08	0.283
Theme: Democracy				0.74	0.62 – 0.89	**0.001**
VeracityTrue:ThemeSocial Justice				1.10	0.86 – 1.41	0.449
VeracityTrue:ThemeDemocracy				1.20	0.93 – 1.54	0.158
Age				0.85	0.55 – 1.31	0.459
Sex: Female	*Reference*			*Reference*		
Sex: Male				0.62	0.29 – 1.33	0.219
Epistemic_curiosity				1.25	0.86 – 1.81	0.237
Education: Brevet	*Reference*			*Reference*		
Education: Bac				0.84	0.02 – 38.66	0.928
Education: Licence				0.59	0.01 – 40.68	0.808
Education: Master				0.57	0.01 – 28.51	0.776
**Random Effects**						
σ^2	3.29			3.29		
τ_{00}	7.34 Subject			7.69 Subject		
τ_{11}	0.39 Subject.JudgmentTrue			0.44 Subject.JudgmentTrue		
	0.31 Subject.Confidence			0.28 Subject.Confidence		
ρ_{01}	0.09			-0.10		
	-0.31			-0.33		
ICC	0.71			0.71		
N	258 Subject			228 Subject		
Observations	12384			10944		
Marginal R^2 / Conditional R^2	0.015 / 0.715			0.033 / 0.719		

We fitted mixed linear models (estimated using REML) to predict subjects' probability to choose to receive additional information with the participant's degree of confidence and its interaction with the veracity judgment (left). We investigated whether the response times effect withstands the inclusion of control variables. Specifically, we included the news veracity, its interaction with the news theme, the response times and the sociodemographics (right). The models included subjects, waves, groups and stimuli presentation order as random effects. Education: Brevet = Below High School degree, Bac = Final High School degree, Licence = Bachelor, Master = Master.

Supplementary Table 16: Degree of confidence in the veracity assessment as determinant of the Willingness-To-Pay for the reception choice

	**WTP**			**WTP**		
Predictors	*Estimates*	*CI*	*p*	*Estimates*	*CI*	*p*
(Intercept)	5.57	4.89 – 6.25	**<0.001**	8.83	2.70 – 14.95	**0.005**
Reception: Reception0	*Reference*			*Reference*		
Reception: Reception1	1.22	0.68 – 1.77	**<0.001**	1.27	0.68 – 1.86	**<0.001**
Confidence	0.54	0.39 – 0.69	**<0.001**	0.49	0.34 – 0.64	**<0.001**
Reception1:Confidence	-0.48	-0.65 – -0.31	**<0.001**	-0.45	-0.63 – -0.27	**<0.001**
Veracity: False	*Reference*			*Reference*		
Veracity: True				0.08	-0.13 – 0.29	0.460
Theme: Ecology	*Reference*			*Reference*		
Theme: Social Justice				0.11	-0.10 – 0.32	0.323
Theme: Democracy				0.01	-0.20 – 0.22	0.950
VeracityTrue:ThemeSocial Justice				-0.32	-0.62 – -0.03	**0.033**
VeracityTrue:ThemeDemocracy				-0.09	-0.38 – 0.21	0.573
Age				-0.13	-0.81 – 0.54	0.703
Sex: Female	*Reference*			*Reference*		
Sex: Male				-0.07	-1.24 – 1.11	0.910
Epistemic_curiosity				0.08	-0.51 – 0.68	0.782
Education: Brevet	*Reference*			*Reference*		
Education: Bac				-3.05	-9.16 – 3.06	0.328
Education: Licence				-2.43	-9.11 – 4.26	0.477
Education: Master				-3.56	-9.79 – 2.66	0.262
**Random Effects**						
σ^2	10.11			10.21		
τ_{00}	29.63 $_{Subject}$			30.26 $_{Subject}$		
τ_{11}	14.65 $_{Subject.Reception1}$			15.46 $_{Subject.Reception1}$		
	0.79 $_{Subject.Confidence}$			0.66 $_{Subject.Confidence}$		
ρ_{01}	-0.48			-0.50		
	0.55			0.55		
ICC	0.74			0.74		
N	258 $_{Subject}$			228 $_{Subject}$		
Observations	12384			10944		
Marginal R^2 / Conditional R^2	0.013 / 0.740			0.020 / 0.741		

We fitted mixed linear models (estimated using REML) to predict subjects' Willingness-To-Pay (WTP) for the reception choice with the participant's degree of confidence and its interaction with the reception choice (left). We investigated whether the response times effect withstands the inclusion of control variables. Specifically, we included the news veracity, its interaction with the news theme, the response times and the sociodemographics (right). The models included subjects, waves, groups and stimuli presentation order as random effects. Education: Brevet = Below High School degree, Bac = Final High School degree, Licence = Bachelor, Master = Master.

6. Moderated Mediation Model

We tested whether the effects of news content imprecision and news content propensity to polarize on reception choices are mediated by the confidence in the veracity evaluation, and moderated by the veracity judgment (Table 2). To that purpose, we performed a moderated mediation model using structural equation modelling. Parameters were bootstrapped, using 10000 repetitions, and confidence intervals were computed using the adjusted bootstrap percentile method at 95% [2.5%, 97.5%]. News content imprecision and propensity to polarize were the predictor variables, with veracity judgment moderating and confidence mediating their effects. Reception choice was the outcome variable. Moderated mediation analyses examined the conditional indirect effect of veracity judgment on the relationship between predictors and the outcome via confidence. We utilized bias-corrected 95% confidence intervals (n = 1000) to assess the significance of conditional indirect effects. This model, with orthogonalized variables, explicitly tested the moderating effect on the predictor-to-mediator path. Veracity judgment was found to moderate the effect on the news content imprecision to confidence path (standardized interaction $\beta = 0.1$, $Z = 2.43$, $p = .015$) as well as the effect on the news content propensity to polarize to confidence path (standardized interaction $\beta = -0.36$, $Z = -8.34$, $p < .001$). We also tested the mediating effect of confidence on the decision to receive extra information about the news (i.e., reception choices). The effect of the mediator confidence on the outcome reception choices was the unique direct effect (standardized interaction $\beta = -0.15$, $Z = -13.96$, $p < .001$). These results reveal the relationships between news content imprecision and polarization, conditional on the veracity judgment, on the choices to receive extra information about the news. Specifically, these relationships are mediated by the negative effect that the confidence in news veracity judgment has on the choices to receive extra information about the news. Confirmatory factor analysis ensured measurement adequacy, with confidence defined as a latent variable predicted by news content propensity to polarize, news content imprecision, veracity judgment, and their interactions. All factor loadings except news content propensity to polarize exceeded 0.6, while composite reliability and average variance extracted surpassed recommended thresholds (0.7 and 0.5, respectively). To evaluate moderated mediation significance, we used an index based on the difference in indirect effects across levels of news veracity, supported by non-zero confidence intervals.

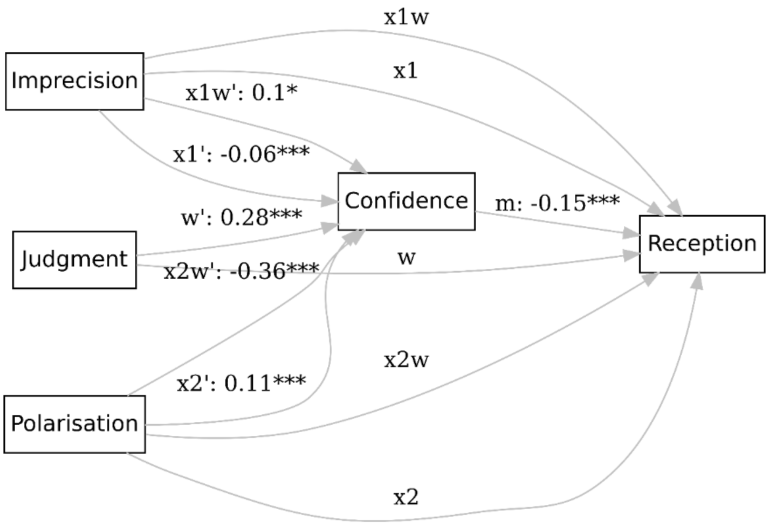

Supplementary Figure 13: Structural equation modelling plot for moderated mediation model predicting the demand for further information. Indirect effects of news content imprecision and news content propensity to polarize, conditional on the veracity judgment, on reception choices via the confidence in the veracity evaluation. The coefficients are standardized. Notes: **p* < .05. ***p* < .01. ****p* < .001.

Supplementary References

1. Pennycook, G., Binnendyk, J., Newton, C. & Rand, D. G. A practical guide to doing behavioral research on fake news and misinformation. *Collabra Psychol.* **7**, 1–13 (2021) doi:10.1525/collabra.25293.
2. Koo, T. K. & Li, M. Y. A Guideline of Selecting and Reporting Intraclass Correlation Coefficients for Reliability Research. *J. Chiropr. Med.* **15**, 155–163 (2016) doi:10.1016/j.jcm.2016.02.012.
3. Barbieri, F., Espinosa Anke, L. & Camacho-Collados, J. *XLM-T: Multilingual Language Models in Twitter for Sentiment Analysis and Beyond*. *Proceedings of the Language Resources and Evaluation Conference* (European Language Resources Association, 2022). doi:https://doi.org/10.48550/arXiv.2104.12250.
4. Wolf, T., Debut, L., Sanh, V., Chaumond, J., Delangue, C., Moi, A., ... & Rush, A. M. Transformers: State-of-the-Art Natural Language Processing. *Proc. 2020 Conf. Empir. methods Nat. Lang. Process. Syst. Demonstr.* 38–45 (2020) doi:10.18653/v1/2020.emnlp-demos.6.
