# Supplementary material for: Metacognition biases information seeking in assessing ambiguous news
Source: Commun Psychol. 2024 Dec 19;2:122. doi: 10.1038/s44271-024-00170-w (PMC11659316; doi:10.1038/s44271-024-00170-w)
Supplement: Supplementary file 3 — Reporting summary [file 44271_2024_170_MOESM3_ESM.pdf]

nature portfolio

Corresponding author(s): COMMSPSYCHOL-24-0220B

Last updated by author(s): Nov 18, 2024

Reporting Summary

Nature Portfolio wishes to improve the reproducibility of the work that we publish. This form provides structure for consistency and transparency in reporting. For further information on Nature Portfolio policies, see our Editorial Policies and the Editorial Policy Checklist.

Statistics

For all statistical analyses, confirm that the following items are present in the figure legend, table legend, main text, or Methods section.

n/a	Confirmed	
☐	☒	The exact sample size (n) for each experimental group/condition, given as a discrete number and unit of measurement
☐	☒	A statement on whether measurements were taken from distinct samples or whether the same sample was measured repeatedly
☐	☒	The statistical test(s) used AND whether they are one- or two-sided<br>*Only common tests should be described solely by name; describe more complex techniques in the Methods section.*
☐	☒	A description of all covariates tested
☐	☒	A description of any assumptions or corrections, such as tests of normality and adjustment for multiple comparisons
☐	☒	A full description of the statistical parameters including central tendency (e.g. means) or other basic estimates (e.g. regression coefficient) AND variation (e.g. standard deviation) or associated estimates of uncertainty (e.g. confidence intervals)
☐	☒	For null hypothesis testing, the test statistic (e.g. F, t, r) with confidence intervals, effect sizes, degrees of freedom and P value noted<br>*Give P values as exact values whenever suitable.*
☐	☒	For Bayesian analysis, information on the choice of priors and Markov chain Monte Carlo settings
☐	☒	For hierarchical and complex designs, identification of the appropriate level for tests and full reporting of outcomes
☐	☒	Estimates of effect sizes (e.g. Cohen's d, Pearson's r), indicating how they were calculated

Our web collection on statistics for biologists contains articles on many of the points above.

Software and code

Policy information about availability of computer code

Data collection	Data was collected on Testable.org using a .csv code format.
Data analysis	Data was analyzed with custom code on MATLAB R2020b, R 3.4.1 and Python 3.11.5.

For manuscripts utilizing custom algorithms or software that are central to the research but not yet described in published literature, software must be made available to editors and reviewers. We strongly encourage code deposition in a community repository (e.g. GitHub). See the Nature Portfolio guidelines for submitting code & software for further information.

Data

Policy information about availability of data

All manuscripts must include a data availability statement. This statement should provide the following information, where applicable:

- Accession codes, unique identifiers, or web links for publicly available datasets
- A description of any restrictions on data availability
- For clinical datasets or third party data, please ensure that the statement adheres to our policy

All raw and processed data used for the main analyses and supplementary information are freely accessible in .csv format via OSF: https://osf.io/436pq/.

Human research participants

Policy information about studies involving human research participants and Sex and Gender in Research.

Reporting on sex and gender	Sex (biological attribute) data was collected by Testable.org as self-reporting measure, as part of their standard procedure for participants' profile creation. To our knowledge, Testable.org didn't make the distinction at the time of the experiment between sex and gender. Sex data was aggregated with task data when collecting experiment data. Gender (shaped by social and cultural circumstances) data was not collected in our post-task questionnaire. Consent has been obtained from all participants as a click-to-accept button following a consent-form screen. Sex data was used in our mixed linear models as sociodemographics data.
Population characteristics	269 participants, aged from 18 to 34 years old, with no history of neurological or psychiatric disorders participated in this online experiment. 258 participants, aged 18 to 34 years, were included in the statistical analyses (131 female participants, 127 male participants).
Recruitment	Participants were recruited from the mailing list of candidates to experimental studies regularly registered in the GATE-Lab pool of experimental subjects, in Lyon, France.
Ethics oversight	The study was approved by an internal ethics review board and complied with the European data protection regulation (GDPR).

Note that full information on the approval of the study protocol must also be provided in the manuscript.

Field-specific reporting

Please select the one below that is the best fit for your research. If you are not sure, read the appropriate sections before making your selection.

☐ Life sciences ☒ Behavioural & social sciences ☐ Ecological, evolutionary & environmental sciences

For a reference copy of the document with all sections, see nature.com/documents/nr-reporting-summary-flat.pdf

Behavioural & social sciences study design

All studies must disclose on these points even when the disclosure is negative.

Study description	We designed an incentivized within-subject experiment in which non-ego relevant news varied in content imprecision and propensity to polarize opinions. Participants were presented with a set of brief news about ecology, democracy and social justice taken from the press that could be either true or false. Participants had to evaluate the veracity of each brief news and quantitatively report their confidence in their judgment on a continuous scale, using a probability elicitation incentivized method. Then, participants had to decide on whether acquiring or not additional information about this news (to be received after the task was performed), and quantitatively report their willingness-to-pay to have their information-seeking choice implemented.
Research sample	269 participants, aged from 18 to 34 years old, with no history of neurological or psychiatric disorders participated in this online experiment. Participants to the experiment were students from business schools and engineering schools regularly registered in the GATE-Lab pool of experimental subjects, in Lyon, France. Two waves of participants took part in the experiment.
Sampling strategy	Data was sampled from users of Testable.org. To determine the sample size, we conducted power analyses for the first wave of data (N=79) and simulated power for sample sizes up to 250 participants. Using Mixed Linear Models (MLMs) to test the confidence hypothesis, we controlled for the veracity judgment and the interaction between news veracity and news theme. With an α level of 0.05, the observed fixed effect of confidence on information-seeking choices ($\beta = -0.15$) aligns with existing literature on confidence-based information-seeking, yielding a power of 0.99. For an estimated fixed effect of $\beta = -0.72$, simulations indicated that a sample size of N = 150 achieves a power of 0.99. For an estimated fixed effect of $\beta = -0.48$, simulations showed that a sample size of N = 200 achieves a power of 0.99. Therefore, our sample size of N = 250 was deemed adequate for testing the study hypotheses and included 5-10% additional participants to account for potential outliers and dropouts.
Data collection	Data were collected on Testable.org.
Timing	Data were collected in two waves. A first one took place with 80 participants in November 2020. A second one with 189 participants spanned from December 2021 to January 2022.
Data exclusions	In total, two participants were excluded from the analyses due to outlying response times ("RT") during news evaluation (one subject: RT = 51.79 ± 26.35; one subject: RT = 1.93 ± 1.31) compared to the mean response time (14.41 ± 8.44). Nine participants were excluded because they did not complete the final questionnaire.
Non-participation	Nine participants dropped out after the experimental task, failing to complete the final questionnaire.
Randomization	Participants were randomly allocated between groups.

Reporting for specific materials, systems and methods

We require information from authors about some types of materials, experimental systems and methods used in many studies. Here, indicate whether each material, system or method listed is relevant to your study. If you are not sure if a list item applies to your research, read the appropriate section before selecting a response.

Materials & experimental systems

n/a	Involved in the study
☒	☐ Antibodies
☒	☐ Eukaryotic cell lines
☒	☐ Palaeontology and archaeology
☒	☐ Animals and other organisms
☒	☐ Clinical data
☒	☐ Dual use research of concern

Methods

n/a	Involved in the study
☒	☐ ChIP-seq
☒	☐ Flow cytometry
☒	☐ MRI-based neuroimaging
